# Association of relative fat mass (RFM) index with diabetes-related mortality and heart disease mortality

**DOI:** 10.1038/s41598-024-81497-6

**Published:** 2024-12-28

**Authors:** Orison O. Woolcott, Edgar Samarasundera, Alicia K. Heath

**Affiliations:** 1 School of Public Health, Faculty of Medicine, Imperial College London, London, UK; 2 Institute for Globally Distributed Open Research and Education (IGDORE), Los Angeles, CA USA

**Keywords:** Abdominal obesity, Diabetes mortality, General obesity, Heart disease mortality, Relative fat mass, Cardiology, Diabetes, Obesity, Cardiovascular diseases, Epidemiology, Public health, Endocrinology, Diabetes, Obesity

## Abstract

Although studies have examined the association of the Relative Fat Mass (RFM, a novel anthropometric index used as a surrogate for whole-body fat percentage) with all-cause mortality, the association of RFM with diabetes-related mortality and heart disease mortality has not been thoroughly investigated. In addition, no study has compared the associations of RFM and waist circumference (a surrogate for intra-abdominal fat) with cause-specific mortality and all-cause mortality. In the present study, we addressed these knowledge gaps. We used data from the US National Health and Nutrition Examination Survey (NHANES) 1999–2018. NHANES III was used for validation. Analyses included 46,535 adults (mean age 46.5 years). During a median follow-up time of 9.7 years, 6,101 participants died (743 from diabetes; 1,514 from heart disease). Compared with BMI and WC, RFM was more strongly associated with diabetes-related mortality in both women and men, adjusting for age, ethnicity, education, and smoking status. All anthropometric measures were similarly strongly associated with heart disease mortality and all-cause mortality. RFM showed greater predictive discrimination of mortality. Similar results were found in NHANES III (*n* = 14,448). In conclusion, RFM is strongly associated with diabetes-related mortality, heart disease mortality, and all-cause mortality, and outperforms conventional adiposity measures for prediction of mortality.

**Supplementary Information:**

The online version contains supplementary material available at 10.1038/s41598-024-81497-6.

## Introduction

Heart disease and diabetes are among the top ten causes of death globally, heart disease being the leading cause of mortality^1^. Obesity is also a sizable public health problem, and it is a major risk factor for developing heart disease and type 2 diabetes^2,3^. Obesity is defined as a body mass index (BMI, calculated as body weight in kilograms divided by the square of height in meters) of 30 kg/m^2^ or more^4^. However, BMI does not distinguish adipose tissue from non-fat tissues^5–7^ nor does it take into account the gender differences in body adiposity as it is known that women tend to have higher body fat than men^5,6^. In addition, BMI cutoffs for obesity classification are not comparable across ethnic groups^8^.

The association of obesity with cardiovascular disease, heart disease mortality, and all-cause mortality is supported by numerous studies^9–14^. In contrast, the association of obesity with diabetes-related mortality has not been thoroughly studied, even though obesity and weight gain are more strongly associated with incident diabetes than with incident coronary heart disease or cerebrovascular disease^15–17^. It is generally accepted that intra-abdominal fat rather than total body fat (assessed using BMI), is a more important risk factor for type 2 diabetes^18,19^ and mortality^20,21^. However, higher measured overall body adiposity is also associated with increased mortality^9–11,22–25^. Thus, it remains unclear whether total body fat or intra-abdominal fat better predicts mortality.

The relative fat mass (RFM) is an index developed to estimate total body fat percentage. It was derived from the ratio of height to waist circumference (WC), as a result of an interest in identifying the simplest combination of anthropometric measures, among several possible candidates, that showed the highest correlation with total body fat percentage in both women and men^26^. RFM has recently been proposed by the American Medical Association as a useful clinical tool to assess overall mortality risk^27^. Although studies have examined the association of RFM with all-cause mortality^28–31^ and cardiovascular mortality^30^, the association of RFM with diabetes-related mortality and heart disease mortality has not been thoroughly investigated^28^. In addition, no study has compared the associations of RFM and WC with cause-specific mortality and all-cause mortality. Moreover, no previous study has compared the discriminative value of RFM and WC for diabetes-related mortality, heart disease mortality, and all-cause mortality. Thus, the aims of this study were to assess the risk of diabetes-related mortality and heart disease mortality in relation to RFM (a surrogate for total body fat percentage), BMI (a commonly utilized surrogate for total body fat), and WC (a surrogate for intra-abdominal fat), and to compare their discriminative ability to predict mortality risk. All-cause mortality was also studied.

## Methods

### Study design and data sources

We analyzed individual-level data from the National Health and Nutrition Examination Survey (NHANES) 1999–2018, a population-based cohort study in the United States. Data from the National Death Index linked to NHANES data provided information on the vital status and cause of death of NHANES participants, with follow-up through December 31, 2019^32^. NHANES III (1988–1994) data was used for validation. Further details of the datasets used are described in Supplementary Methods.

### Participants

Analyses were restricted to adults 20–80 years of age because obesity classification in younger individuals is based on BMI-for-age percentiles^4^ and because from the 2007–2008 NHANES cycle onwards, the upper age limit of participants was 80 years. The unweighted mean response rate among examinees 20–80 years was 66.1% in NHANES 1999–2018 and 71.3% in NHANES III^33^. Participants who were not interviewed and physically examined were excluded from the analysis. Pregnant participants (self-reported or with a positive urine pregnancy test) were also excluded. The proportion of observations with missing data on relevant variables was 6.2% in NHANES 1999–2018 and 5.3% in NHANES III. Thus, a complete-case analysis was performed^34^.

NHANES protocols were approved by the Institutional or Ethics Review Board^35^. The NCHS Research Ethics Review Board also approved the linkage between NHANES survey data and the National Death Index^32^. The present study did not require separate ethics approval because all analyses were performed using publicly available deidentified data only as indicated in the Federal Policy for the Protection of Human Subjects (detailed in 45 CFR part 46), https://www.hhs.gov/ohrp/regulations-and-policy/regulations/common-rule/index.html^36^.

### Outcomes

The primary outcomes were diabetes-related mortality and heart disease mortality. Diabetes-related mortality was defined as deaths in which diabetes was recorded as the underlying cause of death or a contributor factor among multiple causes of death as coded in the National Death Index dataset^37^ to identify all deaths attributable to diabetes, directly or indirectly, since most individuals with diabetes will die as a consequence of diabetes complications^38,39^. All-cause mortality was a secondary outcome. The causes of death available in the National Death Index dataset used for the present analysis were coded using the International Classification of Diseases, Tenth Revision (ICD-10). The codes for diabetes were E10-E14. The codes for heart disease were I00-I09, I11, I13, and I20-I51.

### Assessment of body adiposity

Body weight, height, and WC were measured across survey cycles using standard equipment and procedures performed by trained technologists at mobile examination centers. Weight was recorded in kilograms, and height and WC were measured in centimeters to the nearest 0.1 cm. BMI was calculated as weight in kg divided by the square of height in meters. WC was measured at the level of the upper lateral border of the right ilium^40^, and was used as the surrogate for intra-abdominal fat. RFM was used as the surrogate for total body fat percentage and was calculated as follows:

RFM (%) = 64 − (20 × height/WC) + (12 × sex); sex equals 0 for men and 1 for women; height and WC measured in the same units^26^.

A subset (NHANES 1999–2016) had information on lean body mass (excluding bone mass), as a surrogate for muscle mass, estimated by dual-energy X-ray absorptiometry (DXA). In addition, another subset (NHANES 2005–2006) had information on android fat, as a better surrogate for intra-abdominal fat than waist circumference. NHANES codebook defines android area as the region “around the waist between the mid-point of the lumbar spine and the top of the pelvis”^41^. NHANES DXA data was limited to participants 20–69 years of age.

### Statistical analysis

Cox proportional hazards regression with restricted cubic splines was used to estimate adjusted hazard ratios (aHRs) and 95% confidence intervals (CIs) for mortality associated with each index (RFM, BMI, and WC) treated as a continuous variable, and using standard deviations and quintiles, with the first quintile as the reference. Estimates were adjusted for age (continuous variable), ethnicity, education level, and smoking status. Model covariates were selected based on previous literature^42^. Given the known sex-related differences in body fat percentage^26,43^ and WC^44,45^, analyses were stratified for women and men. Models were tested for possible interactions of age, ethnicity, and smoking status with anthropometric indexes using the Wald test with Bonferroni correction. Details of covariates are provided in Supplementary Methods.

The individual discrimination ability of RFM, BMI, and WC to predict mortality was assessed through analysis of the area under the receiver-operating-characteristic curves by estimating the Harrell’s C-index^46^. Sampling weights, strata, and primary sampling units were included in all analyses. Estimates and standard errors were obtained using Taylor linearization. Details on the assessment of model assumptions and model fit with restricted cubic splines are provided in Supplementary Methods. The number of knots was chosen to identify the best model fit based on the Akaike information criterion (AIC)^47^. Lower AIC values indicate better fit^48^. The location of knots for splines was based on recommended quantiles^49^, depending on the number of knots chosen (Supplementary Table S1). Spline curves for each index were obtained using the “survminer” and “pspline” packages (R 4.3.1; the R Foundation for Statistical Computing, Vienna, Austria, http://www.R-project.org^50^. Spline curves allow to visually obtain hazard ratios at any index value using any other index value as the reference^51^. aHRs and 95% CIs were estimated using the rounded median of each anthropometric index as the reference using the “xbrcspline” command (Stata 14, StataCorp LP, College Station, TX).

Several sensitivity analyses were performed to test the robustness of the findings. To explore possible reverse causation, an analysis was performed excluding individuals with heart disease, stroke, cancer, and renal failure at baseline and an additional analysis excluded the first 3 years of follow-up. We also studied mortality among never-smokers. In addition, for the investigation of the predictive discrimination of anthropometric indexes, we performed sensitivity analyses restricted to individuals without diabetes at baseline. Due to the small number of deaths among participants without diabetes at baseline, HRs for mortality were not estimated in this subsample. Definitions of renal failure and diabetes are described in Supplementary Methods. Since in some cases the cause of death was diabetes and heart disease, we performed a competing risk and multiple competing risk analysis for diabetes mortality, where diabetes was the only cause of death.

To determine whether the association between RFM and diabetes-related mortality was confounded by muscle mass, we performed further analyses additionally adjusted for muscle mass percentage in the subset with available DXA data (NHANES 1999–2016). In addition, to further test the hypothesis that RFM, a surrogate for whole-body fat percentage, rather than intra-abdominal fat, is a better predictor of mortality, we compared the C-index for mortality for RFM and android fat percentage estimated by DXA in the subset with available data (NHANES 2005–2006). Only the first imputation of DXA data was used for these post-hoc analyses.

Harrell’s C-index for each anthropometric index was estimated using the “stcox” and “somersd” commands (Stata 14)^52^ on survey data using untransformed variables. The C-indexes were compared using the Wald test with Bonferroni correction. 95% CIs were obtained using the svy command with the jackknife approach^53^.

We also determined the sensitivity, specificity, the positive and negative predictive values, and the false positive and negative rates of RFM, BMI, and WC. To perform a fair comparison, we identified the cutoffs of baseline RFM, BMI, and WC for diabetes-related mortality, heart disease mortality, and all-cause mortality using the Youden’s index^54^. In addition, we estimated the population attributable fraction to determine the proportion of deaths that would not have occurred if high body adiposity (a modifiable factor) had been eliminated, with the assumption that all other factors would have remained unchangeable and no unaccounted confounding for the exposure was relevant. We performed this analysis separately for each index following the adjusted Cox regressions using a dichotomized exposure (obesity) for sex and cause of death^55^, using published cutoffs for easy interpretation and usability: RFM ≥ 40% for women and ≥ 30% for men^28^; BMI ≥ 30 kg/m^2^ for women and men^4^; and WC > 88 cm for women and > 102 cm for men^56^.

A two-sided P value less than 0.05 was considered statistically significant.

## Results

### Clinical characteristics of NHANES 1999–2018 participants

Among 101,316 participants, in total 51,073 individuals met the inclusion criteria (Supplementary Fig. S1) and were potentially eligible. Of them, 1,469 were excluded because of pregnancy and an additional 3,069 because of missing data (6.2%). The final sample consisted of 46,535 participants (mean age 46.5 years; 50.9% women). During a median follow-up time of 9.7 years [interquartile range (IQR), 5.1–14.7], 6,101 participants died, including 743 deaths from causes related to diabetes and 1,514 from heart disease (Table 1).

**Table 1:** Characteristics of adult participants of NHANES 1999–2018, overall and among those who died from diabetes as the underlying or contributing cause of death*.

Characteristic	All	Women		Men	
		All	Diabetes deaths	All	Diabetes deaths
n	46,535	23,354	319	23,181	424
Age, mean (SD), years	46.5 (16.4)	47.4 (16.4)	65.5 (13.9)	45.7 (16.3)	61.4 (15.7)
Ethnicity, no. (%)					
Mexican American	8,236 (8.2)	4,118 (7.5)	77 (6.8)	4,118 (8.9)	98 (7.5)
European American	20,117 (68.2)	9,924 (68.1)	127 (66.3)	10,193 (68.4)	198 (72.9)
African American	9,889 (11.0)	5,012 (11.7)	83 (16.0)	4,877 (10.3)	88 (11.0)
Other ethnicity	8,293 (12.5)	4,300 (12.7)	32 (10.8)	4,118 (12.4)	40 (8.6)
Body weight, mean (SD), kg	82.1 (21.1)	75.7 (20.0)	82.6 (27.3)	88.7 (20.0)	92.6 (28.6)
Height, mean (SD), cm	168.8 (10.1)	162.0 (6.9)	159.8 (8.0)	175.9 (7.6)	173.2 (10.2)
WC, mean (SD), cm	98.3 (16.3)	95.6 (16.6)	106.8 (21.4)	101.1 (15.6)	110.4 (21.2)
BMI, mean (SD), kg/m^2^^†^	28.7 (6.6)	28.8 (7.3)	32.2 (9.9)	28.6 (5.9)	30.8 (8.6)
RFM, mean (SD), %^‡^	34.9 (8.5)	41.1 (5.9)	45.3 (6.0)	28.4 (5.4)	31.9 (6.2)
Current smoker, no. (%)					
No	36,396 (78.0)	19,118 (80.6)	263 (79.5)	17,278 (75.3)	321 (73.2)
Yes	10,139 (22.0)	4,236 (19.4)	56 (20.5)	5,903 (24.7)	103 (26.8)
Education, no. (%)					
Less than 9th grade	5,443 (5.7)	2,604 (5.4)	78 (15.2)	2,839 (6.0)	114 (16.7)
9th to 11th grade	6,908 (11.2)	3,336 (10.8)	86 (25.4)	3,572 (11.6)	96 (20.1)
12th grade or higher	34,184 (83.1)	17,414 (83.8)	155 (59.4)	16,770 (82.4)	214 (63.2)
Follow-up, median (IQR), years	9.7 (5.1–14.7)	9.8 (5.2–14.8)	7.5 (3.5–11.7)	9.6 (5.0-14.6)	8.3 (4.7–12.3)

*BMI* body mass index,* IQR* interquartile range,* RFM* relative fat mass,* SD* standard deviation,* WC* waist circumference.

*Sample size represents unweighted data. Estimates represent weighted data. Percentages may not total 100 due to rounding. ^†^BMI was calculated as the body weight in kilograms divided by the square of the height in meters. ^‡^RFM was calculated as follows: 64 − (20 × height/waist circumference) + (12 × sex); sex equals 0 for men and 1 for women; height and waist circumference were measured in the same units.

### Association between anthropometric indexes and diabetes-related mortality in NHANES 1999–2018

**Fig. 1:**
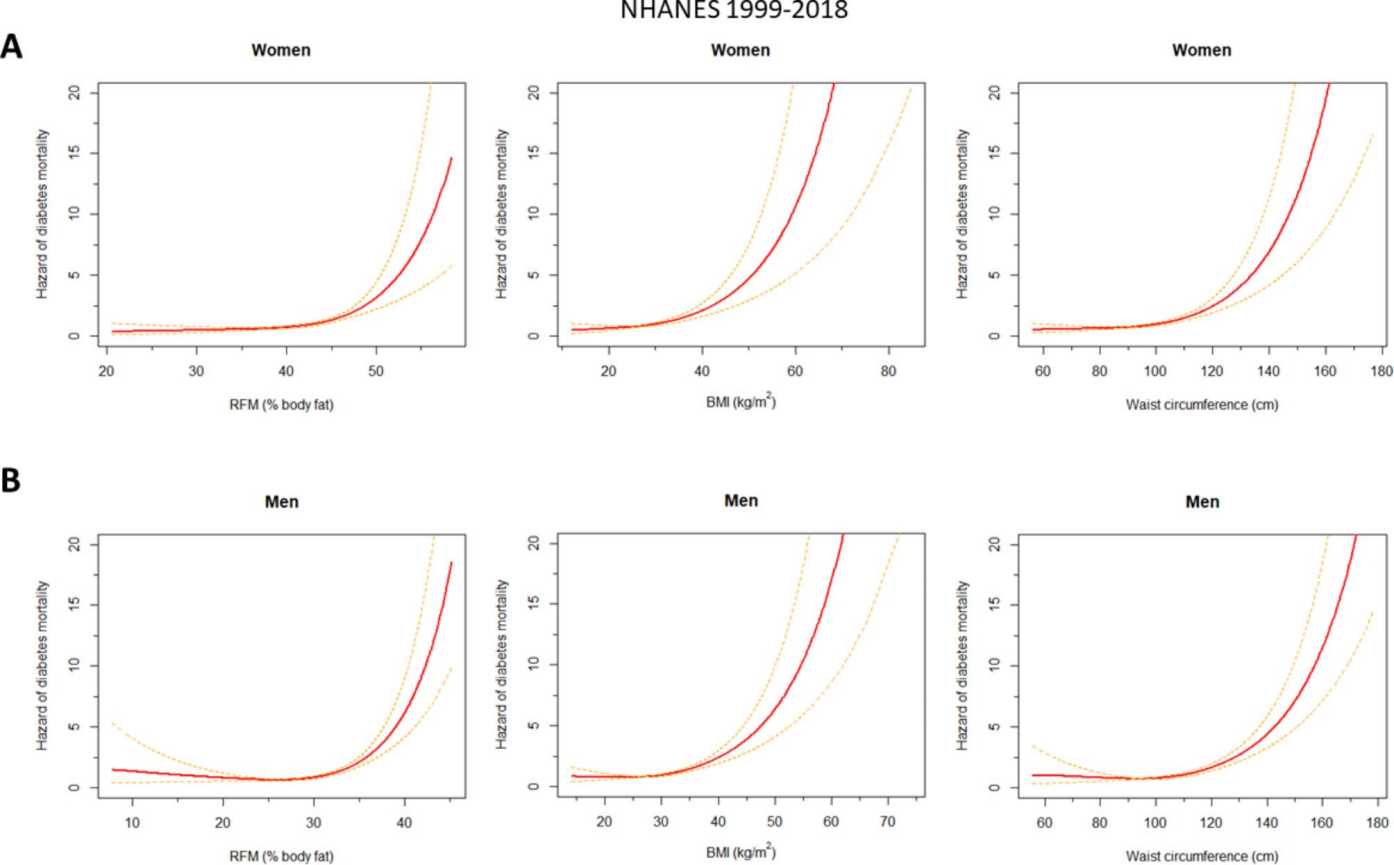
Hazard of diabetes-related mortality in NHANES 1999–2018 according to anthropometric indexes. Plots represent the weighted adjusted hazard, as a measure of risk, of diabetes-related mortality for women (**A**) and men (**B**). All models were adjusted for age, ethnicity, education level, and smoking status. All anthropometric indexes and age were modelled with restricted cubic splines. Note that the curves represent the adjusted hazard (not the hazard ratio) of diabetes-related mortality times a constant (the inverse of the hazard of diabetes-related mortality at the mean linear predictor). As an example, among men, a hazard ratio for diabetes-related mortality can be obtained by dividing the hazard at a given value of relative fat mass (RFM) (e.g., 35%) intersecting the curve (hazard = ~ 2.20) and the hazard at another RFM value arbitrarily chosen as the reference (e.g., 25%) intersecting the same curve (hazard = ~ 0.70). The hazard ratio would be calculated as 2.20/0.70 = 3.14. That is, men with an RFM of 35% have a risk 3.1 times greater than the risk of men with an RFM of 25%. Dashed lines represent the 95% confidence interval.* BMI* body mass index,* RFM* relative fat mass,* WC* waist circumference.

The assumption of proportional hazards was met in all models (Supplementary Figures S2-S4). The linearity assumption was violated (Supplementary Figure S5). RFM showed a better fit when modelled with restricted cubic splines, both among women (*P* = 0.03; splines AIC: 5,373.0 vs. linear AIC: 5,375.9; unweighted data) and among men (*P* = 0.004; splines AIC: 7,278.6 vs. linear AIC: 7,285.6; unweighted data). Spline regressions showed better fit than linear models for BMI (*P* = 0.002) and for WC (*P* = 0.022) among men but were similar to linear models among women (BMI: *P* = 0.06; WC: *P* = 0.07). Thus, for comparison purposes, splines were used. All anthropometric indexes showed a monotonic positive association with diabetes-related mortality among women and a weakly non-monotonic association among men (Fig. 1). Similar findings were observed when quintiles were used (Supplementary Figure S6, Supplementary Table S2). Exclusion of individuals with heart disease, stroke, cancer, and renal failure at baseline resulted in a slight shift to the left of the association curves among women and men (Supplementary Figure S7). Exclusion of the first 3 years of follow-up resulted in a modest shift to the right of the association curves among women and a slight shift to the left among men (Supplementary Figure S8). The association curves were shifted to the left when the analysis was restricted to men who never smoked, but no differences were observed among women who never smoked (Supplementary Figure S9). Table 2 shows aHRs of diabetes-related mortality relative to the 25th percentile of the corresponding index (arbitrarily chosen reference category). For example, women with an RFM of 43% had a 59% greater risk of diabetes-related mortality than women with an RFM of 37% (aHR: 1.59, 95% CI; 1.28–1.99). Overall, RFM was more strongly associated with diabetes-related mortality compared with BMI and WC, in both women and men (Table 2). This superiority was not apparent when analysis was restricted to quintiles (Supplementary Table S2). When the model for RFM was further adjusted for muscle mass percentage in a subset with available DXA data, the association of RFM with diabetes-related mortality remained (Supplementary Table S3). However, the estimates for women should be interpreted with caution due to the small number of events in the first quintile. For example, men in the fifth quintile had 2.7 times greater risk than those in the first quintile (aHR: 2.70, 1.02–7.20). In contrast, women in the fifth quintile had 20 times greater risk than those in the first quintile (aHR: 20.10, 3.91–103.30).

**Table 2:** Associations of relative fat mass, body mass index and waist circumference with diabetes-related mortality among adult participants of NHANES 1999–2018*.

	RFM^†^, %	Hazard ratio (95% CI)	BMI^‡^, kg/m^2^	Hazard ratio (95% CI)	WC, cm	Hazard ratio (95% CI)
Women (*n* = 23,354)						
	25	0.70 (0.33–1.48)	11	0.65 (0.35–1.24)	51	1.44 (0.51–4.06)
	28	0.76 (0.43–1.33)	14	0.73 (0.45–1.17)	59	1.32 (0.61–2.86)
	31	0.83 (0.57–1.20)	17	0.81 (0.59–1.11)	67	1.20 (0.72–2.01)
	34	0.90 (0.75–1.09)	20	0.90 (0.77–1.05)	75	1.10 (0.85–1.42)
25th percentile	37	1.00 (Reference)	23	1.00 (Reference)	83	1.00 (Reference)
	40	1.18 (1.03–1.37)	26	1.13 (0.98–1.30)	91	0.93 (0.73–1.19)
	43	1.59 (1.28–1.99)	29	1.31 (1.03–1.67)	99	0.97 (0.63–1.48)
	46	2.50 (1.92–3.27)	32	1.58 (1.18–2.12)	107	1.18 (0.72–1.95)
	49	4.27 (2.97–6.12)	35	1.97 (1.45–2.69)	115	1.65 (0.99–2.73)
	52	7.37 (4.47–12.16)	38	2.50 (1.82–3.45)	123	2.41 (1.48–3.93)
Men (n = 23,181)						
	13	1.75 (0.70–4.36)	13	1.05 (0.47–2.34)	58	1.42 (0.51–3.95)
	16	1.51 (0.76–2.97)	16	1.04 (0.57–1.88)	66	1.30 (0.60–2.78)
	19	1.30 (0.83–2.03)	19	1.02 (0.69–1.51)	74	1.18 (0.71–1.96)
	22	1.12 (0.90–1.39)	22	1.00 (0.83–1.22)	82	1.08 (0.84–1.38)
25th percentile	25	1.00 (Reference)	25	1.00 (Reference)	90	1.00 (Reference)
	28	1.05 (0.90–1.23)	28	1.07 (0.93–1.23)	98	1.02 (0.84–1.23)
	31	1.47 (1.17–1.84)	31	1.28 (1.05–1.57)	106	1.22 (0.92–1.62)
	34	2.58 (1.99–3.35)	34	1.67 (1.34–2.07)	114	1.68 (1.24–2.26)
	37	4.87 (3.48–6.82)	37	2.23 (1.76–2.83)	122	2.45 (1.81–3.30)
	40	9.20 (5.91–14.32)	40	3.00 (2.29–3.92)	130	3.60 (2.62–4.94)

*BMI* body mass index,* CI* confidence interval,* RFM* relative fat mass, * WC* waist circumference.

*Estimates represent adjusted hazard ratios obtained from Cox regression models with RFM, BMI, and WC modelled using restricted cubic splines. Hazard ratios were adjusted for age (as restricted cubic splines), ethnicity, education level, and smoking status. The rounded weighted 25th percentile of each anthropometric index was arbitrarily chosen as the reference. Index values are shown in increments of approximately 0.5 of their standard deviation to facilitate comparison. ^†^RFM was calculated as follows: 64 − (20 × height/waist circumference) + (12 × sex); sex equals 0 for men and 1 for women; height and waist circumference were measured in the same units. ^‡^BMI was calculated as body weight in kilograms divided by the square of the height in meters.

Among men, there was suggestion of an interaction between RFM and smoking status (*P* = 0.04). Current smokers appeared to have a slightly higher risk of diabetes-related mortality at lower RFM values (J-shaped curve), which was not observed in never/former smokers (Supplementary Figure S10). Among women, there was evidence of an interaction between WC and age (*P* = 0.02). The positive association between WC and diabetes-related mortality was stronger among women younger than 60 years of age than in women aged ≥ 60 years, particularly at higher WC values (Supplementary Figure S11).

The competing risk analyses for diabetes mortality showed very similar findings to the standard Cox regression analysis (Supplementary Table S4). The findings in Supplementary Table S4 also show that RFM, BMI, and WC were associated with diabetes mortality, when diabetes was the only cause of death.

### Association of anthropometric indexes with heart disease mortality and all-cause mortality in NHANES 1999–2018

The assumption of proportionality across models were met. RFM and WC showed monotonic positive associations with heart disease mortality among women. Non-monotonic associations between RFM, BMI and WC and heart disease mortality were observed among men, and for BMI among women (Table 3, Supplementary Figure S12). The association between anthropometric indexes and all-cause mortality showed a non-monotonic relationship across all indexes, except for RFM and WC among women (Table 4, Supplementary Figure S13).

**Table 3:** Associations of relative fat mass, body mass index, and waist circumference with heart disease mortality among adult participants of NHANES 1999–2018*.

	RFM^†^, %	Hazard ratio (95% CI)	BMI^‡^, kg/m^2^	Hazard ratio (95% CI)	WC, cm	Hazard ratio (95% CI)
Women (23,355)						
	25	0.69 (0.27–1.76)	11	2.13 (0.85–5.33)	51	0.84 (0.30–2.31)
	28	0.76 (0.38–1.51)	14	1.75 (0.89–3.47)	59	0.88 (0.41–1.86)
	31	0.84 (0.54–1.3)	17	1.45 (0.92–2.26)	67	0.92 (0.56–1.50)
	34	0.92 (0.76–1.12)	20	1.19 (0.96–1.48)	75	0.96 (0.77–1.21)
25th percentile	37	1.00 (Reference)	23	1.00 (Reference)	83	1.00 (Reference)
	40	1.05 (0.94–1.16)	26	0.92 (0.82–1.04)	91	1.02 (0.91–1.13)
	43	1.07 (0.87–1.33)	29	0.98 (0.84–1.15)	99	1.03 (0.86–1.23)
	46	1.27 (0.96–1.69)	32	1.13 (0.91–1.4)	107	1.15 (0.90–1.48)
	49	1.83 (1.40–2.40)	35	1.32 (1.02–1.71)	115	1.44 (1.11–1.87)
	52	2.83 (2.00–4.00)	38	1.56 (1.20–2.05)	123	1.93 (1.48–2.52)
Men (23,181)						
	13	1.53 (0.86–2.72)	13	1.45 (0.88–2.39)	58	1.35 (0.76–2.41)
	16	1.36 (0.89–2.10)	16	1.32 (0.91–1.91)	66	1.25 (0.81–1.93)
	19	1.22 (0.92–1.62)	19	1.20 (0.94–1.53)	74	1.16 (0.87–1.54)
	22	1.09 (0.95–1.25)	22	1.09 (0.96–1.23)	82	1.07 (0.93–1.23)
25th percentile	25	1.00 (Reference)	25	1.00 (Reference)	90	1.00 (Reference)
	28	1.02 (0.92–1.12)	28	1.00 (0.92–1.09)	98	1.00 (0.90–1.11)
	31	1.24 (1.08–1.42)	31	1.13 (1.01–1.28)	106	1.12 (0.96–1.30)
	34	1.76 (1.47–2.10)	34	1.41 (1.23–1.62)	114	1.38 (1.17–1.63)
	37	2.60 (2.02–3.36)	37	1.82 (1.53–2.15)	122	1.78 (1.48–2.16)
	40	3.86 (2.72–5.48)	40	2.34 (1.89–2.91)	130	2.31 (1.83–2.93)

*BMI* body mass index,* CI* confidence interval,* RFM* relative fat mass,* WC* waist circumference.

*Estimates represent adjusted hazard ratios obtained from Cox regression models for RFM, BMI, and WC modelled using restricted cubic splines. Hazard ratios were adjusted for age (as restricted cubic splines), ethnicity, education level, and smoking status. The rounded weighted 25th percentile of each index was arbitrarily chosen as the reference. Index values are shown in increments of approximately 0.5 of their standard deviation to facilitate comparison. ^†^RFM was calculated as follows: 64 − (20 × height/waist circumference) + (12 × sex); sex equals 0 for men and 1 for women; height and waist circumference were measured in the same units. ^‡^BMI was calculated as body weight in kilograms divided by the square of the height in meters.

**Table 4:** Associations of relative fat mass, body mass index, and waist circumference with all-cause mortality among adult participants of NHANES 1999–2018*.

	RFM^†^, %	Hazard ratio (95% CI)	BMI^‡^, kg/m^2^	Hazard ratio (95% CI)	WC, cm	Hazard ratio (95% CI)
Women (23,355)						
	25	1.03 (0.66–1.62)	11	2.92 (1.66–5.13)	51	0.92 (0.49–1.75)
	28	1.02 (0.74–1.41)	14	2.21 (1.46–3.36)	59	0.95 (0.59–1.51)
	31	1.01 (0.83–1.23)	17	1.68 (1.28–2.20)	67	0.97 (0.72–1.31)
	34	1.00 (0.93–1.08)	20	1.27 (1.13–1.44)	75	1.00 (0.88–1.13)
25th percentile	37	1.00 (Reference)	23	1.00 (Reference)	83	1.00 (Reference)
	40	1.01 (0.92–1.10)	26	0.93 (0.87-1.00)	91	0.94 (0.85–1.03)
	43	1.01 (0.86–1.19)	29	0.98 (0.85–1.13)	99	0.92 (0.79–1.06)
	46	1.07 (0.93–1.24)	32	1.04 (0.91–1.18)	107	1.01 (0.88–1.15)
	49	1.39 (1.21–1.60)	35	1.12 (0.98–1.28)	115	1.18 (1.03–1.35)
	52	1.99 (1.61–2.46)	38	1.24 (1.08–1.43)	123	1.43 (1.25–1.65)
Men (23,181)						
	13	1.98 (1.29–3.04)	13	3.99 (2.40–6.66)	58	2.28 (1.22–4.24)
	16	1.66 (1.23–2.25)	16	2.74 (1.91–3.94)	66	1.84 (1.18–2.88)
	19	1.40 (1.17–1.67)	19	1.88 (1.52–2.34)	74	1.49 (1.14–1.95)
	22	1.17 (1.10–1.26)	22	1.3 (1.20–1.40)	82	1.21 (1.09–1.34)
25th percentile	25	1.00 (Reference)	25	1.00 (Reference)	90	1.00 (Reference)
	28	0.89 (0.79–0.99)	28	1.00 (0.89–1.11)	98	0.89 (0.79-1.00)
	31	0.98 (0.87–1.11)	31	1.15 (1.04–1.28)	106	0.97 (0.85–1.11)
	34	1.29 (1.14–1.47)	34	1.35 (1.20–1.53)	114	1.16 (1.02–1.32)
	37	1.78 (1.54–2.06)	37	1.58 (1.39–1.79)	122	1.40 (1.23–1.58)
	40	2.46 (1.95–3.10)	40	1.83 (1.60–2.11)	130	1.68 (1.45–1.93)

*BMI* body mass index,* CI* confidence interval,* RFM* relative fat mass,* WC* waist circumference.

*Estimates represent adjusted hazard ratios obtained from Cox regression models for RFM, BMI, and WC modelled using restricted cubic splines. Hazard ratios were adjusted for age (as restricted cubic splines), ethnicity, education level, and smoking status. The rounded weighted 25th percentile of each index was arbitrarily chosen as the reference. Index values are shown in increments of approximately 0.5 of their standard deviation to facilitate comparison. ^†^RFM was calculated as follows: 64 − (20 × height/waist circumference) + (12 × sex); sex equals 0 for men and 1 for women; height and waist circumference were measured in the same units. ^‡^BMI was calculated as body weight in kilograms divided by the square of the height in meters.

### Predictive discrimination of mortality in NHANES 1999–2018

Compared with BMI and WC, RFM showed a better discriminative value for diabetes-related mortality among women and men (Supplementary Table S5). Among women, the C-index was 0.73 (95% CI, 0.69–0.77) for RFM, 0.63 (95% CI, 0.58–0.68) for BMI (*P* < 0.001 vs. RFM, with Bonferroni correction) and 0.71 (95% CI, 0.67–0.75) for WC (*P* < 0.001 vs. RFM). Among men, the C-index was 0.71 (95% CI, 0.67–0.75) for RFM, 0.59 (95% CI, 0.55–0.64) for BMI (*P* < 0.001 vs. RFM) and 0.67 (95% CI, 0.63–0.71) for WC (*P* < 0.001 vs. RFM). Analysis restricted to individuals without heart disease, stroke, cancer, and renal failure at baseline, restricted to those with a follow-up longer than 3 years, or restricted to never-smokers resulted in similar differences across indexes. When excluding participants with diabetes at baseline, the C-index was lower across anthropometric indexes among women but not men.

RFM, compared with BMI and WC, showed slightly greater predictive discrimination of heart disease mortality among women and men (Supplementary Table S6), but this superiority was more modest for predicting all-cause mortality (Supplementary Table S7). Similar findings were obtained when using DXA as a direct measure of body fat (Supplementary Table S8).

The diagnostic performance based on sensitivity, specificity, and other metrics of RFM, BMI, and WC are shown in Table 5. Overall, waist circumference-based anthropometric measures performed better than BMI. The proportion of preventable diabetes-related mortality attributed to RFM-diagnosed obesity was 45.1% (95% CI, 22.1–61.3%) among women and 38.5% (95% CI, 26.4–48.6%) among men. The proportion of preventable heart disease mortality attributed to RFM-diagnosed obesity was of smaller magnitude (20.0% for women and 21.4% for men) and less for all-cause mortality. The population attributable fractions varied considerably depending on the index used for obesity diagnosis (Table 6).

**Table 5:** Diagnostic performance of the relative fat mass index, body mass index, and waist circumference for mortality* NHANES 1999–2018.

	Women (*n* = 23,354)							Men (*n* = 23,181)						
	Sens %	Spec %	PPV %	NPV %	FPR %	FNR %	Cutoff^†^	Sens %	Spec %	PPV %	NPV %	FPR %	FNR %	Cutoff^†^
Diabetes-related mortality														
RFM	66.2	62.7	1.7	99.5	37.3	33.8	43.5	53.6	77.6	2.8	99.3	22.4	46.4	32.4
BMI	44.7	73.5	1.6	99.3	26.5	55.3	32.3	48.1	67.5	1.8	99.1	32.5	51.9	30.2
WC	54.3	72.5	1.8	99.4	27.5	45.7	104.2	58.3	69.3	2.2	99.3	30.7	41.7	107.1
Heart disease mortality														
RFM	65.7	53.9	2.8	98.7	46.1	34.3	42.1	58.0	64.4	4.3	98.2	35.6	42.0	30.5
BMI	71.8	35.7	2.2	98.4	64.3	28.3	25.0	47.4	61.5	3.3	97.7	38.5	52.6	29.2
WC	63.1	51.5	2.5	98.6	48.5	36.9	94.0	59.9	58.2	3.8	98.2	41.8	40.1	102.5
All-cause mortality														
RFM	60.5	53.0	11.1	93.3	47.0	39.5	41.8	51.1	65.5	15.2	91.7	34.5	48.9	30.5
BMI	70.6	32.3	9.2	91.9	67.7	29.4	24.5	37.5	65.1	11.5	89.6	35.0	62.5	29.7
WC	53.9	56.1	10.6	92.6	43.9	46.2	95.8	54.1	58.9	13.7	91.4	41.1	46.0	102.4

*All estimates were obtained using the Youden’s index to select the index cutoffs based on weighted data.* BMI* body mass index,* FNR* false negative rate,* FPR* false positive rate,* NPV* negative predictive value,* PPV* positive predictive value,* RFM* relative fat mass,* Sens* sensitivity,* Spec* specificity, *WC* waist circumference.

^†^Cutoffs are expressed in % for RFM, kg/m^2^ for BMI, and cm for WC. The number of deaths among women and men, respectively, is as follows: diabetes-related mortality, 319 and 424; heart disease mortality, 596 and 918; all-cause mortality, 2,551 and 3,550.

**Table 6:** Population attributable fraction for mortality according to anthropometric indexes* NHANES 1999–2018.

	Women (*n* = 23,354)			Men (*n* = 23,181)		
	Prevalence of exposure^†^, %	Hazard ratio (95% CI)	Population attributable fraction^‡^, %	Prevalence of exposure^†^, %	Hazard ratio (95% CI)	Population attributable fraction^‡^, %
Diabetes-related mortality						
RFM	59.1	2.17 (1.43–3.30)	45.1 (26.5–59.0)	40.5	2.30 (1.76–3.02)	38.5 (30.1–45.9)
BMI	36.9	2.11 (1.47–3.03)	28.3 (18.5–36.9)	33.7	2.27 (1.76–2.92)	27.7 (22.0–33.0)
WC	63.5	1.98 (1.38–2.83)	41.5 (24.3–54.8)	43.8	2.16 (1.66–2.81)	36.6 (27.7–44.4)
Heart disease mortality						
RFM	59.1	1.35 (1.10–1.66)	20.0 (7.7–30.7)	40.5	1.54 (1.30–1.83)	21.4 (14.4–27.8)
BMI	36.9	1.38 (1.16–1.65)	11.8 (6.2–17.1)	33.7	1.55 (1.31–1.84)	14.4 (9.8–18.8)
WC	63.5	1.21 (1.00–1.46)	13.4 (0.5–24.6)	43.8	1.42 (1.22–1.65)	18.1 (11.4–24.4)
All-cause mortality						
RFM	59.1	1.09 (0.98–1.21)	5.9 (-1.0 to 12.4)	40.5	1.25 (1.15–1.36)	10.7 (7.0–14.3)
BMI	36.9	1.12 (1.02–1.23)	3.9 (0.7–7.0)	33.7	1.24 (1.13–1.35)	6.8 (4.3–9.2)
WC	63.5	1.01 (0.92–1.12)	0.9 (-6.2 to 7.5)	43.8	1.16 (1.06–1.28)	7.8 (3.3–12.0)

*BMI* body mass index,* CI* confidence interval,* RFM* relative fat mass,* WC* waist circumference.

*All estimates are weighted. ^†^Prevalence was estimated from the NHANES 1999–2018 dataset. ^‡^Estimates among participants without obesity defined based on published cutoffs (if RFM < 40% for women and < 30% for men; BMI < 30 kg/m^2^ for women and men; and WC ≤ 102 cm for men and ≤ 88 cm for women. The population attributable fraction was obtained following a Cox regression adjusted for confounders (age, ethnicity, education level, and smoking status). RFM, BMI, WC, and age were modelled as continuous variables.

### Clinical characteristics of NHANES III participants

The final sample for NHANES III analyses consisted of 14,448 participants (median age 41 years; 51.1% women) (Supplementary Fig. S1 and Supplementary Table S9). During a median follow-up time of 27.1 years (IQR, 23.0-28.8). 5,673 participants died, including 727 participants from causes related to diabetes and 1,608 from heart disease.

### Association between anthropometric indexes and mortality in NHANES III

The assumption of proportional hazards was met across models using this validation dataset. RFM, BMI, and WC showed positive associations with diabetes-related mortality among women and men (Supplementary Figure S14). Exclusion of the first 3 years of follow-up did not alter the associations (Supplementary Figure S15). Exclusion of individuals with chronic disease at baseline resulted in a shift to the left of the association curves among men but not women (Supplementary Figure S16). Supplementary Table S10 shows aHRs of diabetes-related mortality relative to the 25th percentile of the corresponding index. RFM was slightly more strongly associated with diabetes-related mortality compared with BMI and WC.

A J-shaped association of BMI with heart disease mortality (Supplementary Figure S17) and all-cause mortality (Supplementary Figure S18) was observed. RFM, BMI, and WC showed strong associations with heart disease mortality (Supplementary Table S11) and all-cause mortality (Supplementary Table S12).

### Predictive discrimination of mortality in NHANES III

RFM showed slightly greater predictive discrimination of diabetes-related mortality than BMI among women and men. RFM also showed greater predictive discrimination of diabetes-related mortality than WC among men (Supplementary Table S13). RFM was superior to BMI and WC for predictive discrimination of heart disease mortality and all-cause mortality (Supplementary Table S14).

## Discussion

In this population-based study, RFM was more strongly associated with diabetes-related mortality compared with BMI and WC in both women and men. All anthropometric measures were similarly strongly associated with heart disease mortality and all-cause mortality. However, the predictive discrimination ability of RFM for diabetes-related mortality, heart disease mortality, and all-cause mortality was superior to that of BMI and WC among women and men.

Numerous studies have concluded that abdominal fat rather than total body fat is a more important risk factor for cardiovascular disease, diabetes, and all-cause mortality^20,21,57–62^. However, previous studies utilized BMI as a surrogate for total body fat. BMI is known to have important limitations to discriminate body fat from muscle mass^5–7^. In the present study, RFM was utilised as a surrogate for total body fat^26^. RFM is a validated index with greater accuracy to estimate total body fat percentage and greater predictive discrimination of high body fat percentage than BMI-based models among men, and similar to BMI-based models among women^26^.

The associations of BMI and WC with diabetes-related mortality shown in the present study contrast to those from a previous study restricted to Mexican Americans^63^. The authors reported a direct association between waist-to-height ratio and diabetes-related mortality, among women and men, but no association for BMI or WC. Possible explanations for these differences include the relatively small sample size (*n* = 5,849 participants and 107 diabetes-related deaths), different covariates included in the model, and the type of model used (quadratic) in the previous study. Another study reported a strong positive association between BMI at age 17 years and diabetes mortality in midlife^64^.

The findings of the present study suggest that total body fat estimated by RFM, rather than intra-abdominal fat, is a better predictor of diabetes-related mortality, heart disease mortality, and all-cause mortality. However, unanswered questions are how much WC is a surrogate for total body fat and how much RFM is a surrogate for intra-abdominal fat, subcutaneous abdominal fat, and non-abdominal fat. Certainly, all waist-based indexes correlate with intra-abdominal fat^65,66^, including RFM^26^. However, RFM has been shown to have a lower prediction error for whole-body fat percentage than for trunk fat percentage^26^. One speculation about the additional power of RFM over WC to predict diabetes-related mortality and heart disease mortality, RFM being also based on WC, is that RFM also “picks” detrimental non-abdominal fat such as epicardial or pericardial fat, which are also associated with cardiometabolic disease^12^. Recent studies have shown that RFM predicts incident diabetes, atrial fibrillation, heart failure, and coronary artery disease^67,68^. Our findings using DXA data also suggest that total body fat, rather than intra-abdominal fat, is a better predictor of diabetes-related mortality, heart disease mortality, and all-cause mortality.

The possible confounding effect of muscle mass on the association between body adiposity and mortality should not be ignored^69,70^. High fat-free mass, usually accompanied by lower fat mass, is associated with lower mortality, and vice versa^11,71,72^. A recent study showed that the positive association between RFM and all-cause mortality was stronger after adjustment for 24-h urinary creatinine excretion, a marker of total muscle mass^31^. In our study, the associations of relative fat mass, body mass index, and waist circumference with diabetes-related mortality were stronger in women after further adjustment for muscle mass percentage estimated using DXA. The commonly reported U- or J-shaped association between common anthropometrics and mortality was not found in the present study when examining diabetes-related mortality among women, as previously reported^30^, but it was evident for RFM among male current smokers. J-shaped curves were more often observed in the association between anthropometric indexes and heart disease mortality and all-cause mortality, particularly for BMI. Others have shown a J-shaped association between BMI and all-cause mortality among never-smokers^73^ or regardless of smoking status^74^.

In our study, compared with BMI and WC, RFM showed greater predictive discrimination of mortality among both women and men. Data from the Moli-sani study showed that RFM was associated with all-cause mortality among men but not among women^29^. In contrast, data from the PREVEND study showed that RFM was associated with all-cause mortality among women but not among men, unless adjusted for a surrogate for muscle mass^31^. Possible explanations for these discrepancies are differences in baseline characteristics of the populations studied, lifestyle, and adjustment for confounders. Longitudinal studies with repeated anthropometric measurements are needed to better capture their association with mortality. Although RFM, BMI, and WC were strongly associated with diabetes-related mortality, heart disease mortality, and all-cause mortality, only RFM adequately discriminated diabetes-related mortality among both women and men, as indicated by a C-index > 0.70. WC adequately discriminated diabetes-related mortality among women only. Discrimination was more modest for heart disease mortality and all-cause mortality.

Our study has several strengths. First, the participants are representative of the non-institutionalized adult population of the U.S., and the sample size was large enough to obtain reliable estimates. Second, the findings were validated in an independent cohort. Third, regression with restricted cubic splines was used as it provides a better indication of the shape of association^49^. Fourth, several sensitivity analyses were also conducted to confirm the robustness of the results.

The study has some limitations. First, RFM, BMI, and WC were measured only at a single point in time, at baseline. However, our analysis used prospectively collected data on the outcomes (mortality). Second, no information was available on the type of diabetes. However, deaths attributed to type 1 diabetes have a distinct pathophysiology and usually not strongly associated with obesity as in the case of type 2 diabetes^75^. In addition, many participants may have developed diabetes or other comorbidities during the follow-up period, which may have influenced our estimates. This is a limitation of NHANES data. Third, the possible influence of diabetes care on diabetes-related mortality and heart disease mortality could not be determined. Fourth, the National Death Index dataset used does not provide specific information on diabetes complications that may have led to diabetes mortality or information on the specific cause of heart disease mortality (e.g., coronary heart disease, pericarditis, arrhythmias). Fifth, the NHANES III data used for validation was from the same country and a different period. It would be useful to validate the findings in studies with data from the same period (1999–2018) in other countries. We should also consider the possibility of inaccurate attribution of the underlying cause of death in some cases. Finally, given the limited number of events (deaths) per 2-year cycle throughout NHANES, we were not able to evaluate potential temporal trends in the associations of anthropometric measurements with diabetes-related mortality and heart disease mortality.

Our findings provide evidence that RFM can be an alternative tool to BMI to better predict diabetes-related mortality, heart disease mortality and all-cause mortality. Our data also support the value of waist-based anthropometrics to predict mortality^20,21,28,57–62^. Moreover, our study estimated that under certain assumptions, 45.1% of all diabetes-related deaths in women and 38.5% in men and 20% of all heart disease deaths in women and 21.4% in men would have been prevented if all participants had a an RFM below 40% for women and below 30% for men; findings that support the need for further public health interventions. However, the contribution of several other modifiable risk factors for death should also be considered. In large population-based studies, when direct measures of body composition are not feasible, the use of RFM could facilitate the re-evaluation of the association of total body fat with all-cause mortality and cause-specific mortality (and other health outcomes) as previous association studies have largely relied on BMI as a proxy of total body fat. In recent years, some medical associations and professional societies have recommended the use of WC in clinical practice^27,76–78^. However, the clinical use of WC remains a challenge as concerns exist on the method and location of measurement^79^. Thus, guidelines on technical aspects and training of healthcare professionals on WC measurement should be prioritized.

In conclusion, in the general adult population of the U.S., RFM was strongly positively associated with diabetes-related mortality, heart disease mortality, and all-cause mortality, and was superior to BMI and WC as a discriminative tool to predict mortality risk among both women and men.

## Electronic supplementary material

Below is the link to the electronic supplementary material.

Supplementary Material 1

## Data Availability

All data utilised for analysis in this study are fully and publicly available at: https://wwwn.cdc.gov/nchs/nhanes/.
