# Supplementary material for: Association of relative fat mass (RFM) index with diabetes-related mortality and heart disease mortality
Source: Sci Rep. 2024 Dec 28;14:30823. doi: 10.1038/s41598-024-81497-6 (PMC11681238; doi:10.1038/s41598-024-81497-6)
Supplement: Supplementary file 1 — Supplementary Material 1 [file 41598_2024_81497_MOESM1_ESM.pdf]

Supplemental Material

Association of relative fat mass (RFM) index with diabetes-related mortality and heart disease mortality

Orison O. Woolcott, Edgar Samarasundera, Alicia K. Heath

Contents

Supplementary Methods 3

Supplementary Table S1. Number of knots used for cubic spline regression models. 4

Supplementary Table S2. Associations of relative fat mass, body mass index, and waist circumference with diabetes-related mortality, by quintiles. NHANES 1999-2018. 5

Supplementary Table S3. Associations of relative fat mass, body mass index, and waist circumference with diabetes-related mortality, by quintiles, after further adjustment for muscle mass percentage. NHANES 1999-2006.* 6

Supplementary Table S4. Competing risk regression analysis for diabetes mortality where diabetes was the underlying cause of death. NHANES 1999-2014. 7

Supplementary Table S5. Discriminatory prediction of diabetes-related mortality among adult participants of NHANES 1999-2018. 8

Supplementary Table S6. Discriminatory prediction of heart disease mortality among adult participants of NHANES 1999-2018. 9

Supplementary Table S7. Discriminatory prediction of all-cause mortality among adult participants of NHANES 1999-2018. 10

Supplementary Table S8. Discriminatory prediction of RFM, DXA-measured whole body fat, and intra-abdominal fat for mortality among adult participants of NHANES 2005-2006.* 11

Supplementary Table S9. Characteristics of adult participants of NHANES III (1988-1994), overall and among those who died from diabetes as the underlying or contributing cause of death. 12

Supplementary Table S10. Associations of relative fat mass, body mass index and waist circumference with diabetes-related mortality among adult participants of NHANES III (1988-1994) 13

Supplementary Table S11. Associations of relative fat mass, body mass index, and waist circumference with heart disease mortality among adult participants of NHANES III (1988-1994). 14

Supplementary Table S12. Associations of relative fat mass, body mass index, and waist circumference with all-cause mortality among adult participants of NHANES III (1988-1994). 15

Supplementary Table S13. Discriminatory prediction of diabetes-related mortality among adult participants of NHANES III (1988-1994). 16

Supplementary Table S14. Discriminatory prediction of heart disease mortality and all-cause mortality among adult participants of NHANES III (1988-1994). 17

Supplementary Figure S1. Selection of study participants. 18

Supplementary Figure S2. Assumption of proportionality – RFM – NHANES 1999-2018. 19

Supplementary Figure S3. Assessment of assumption of proportionality – BMI – NHANES 1999-2018. 20

Supplementary Figure S4. Assessment of assumption of proportionality – WC – NHANES 1999-2018. 21

Supplementary Figure S5. Assessment of assumption of linearity – NHANES 1999-2018. 22
Supplementary Figure S6. Associations of relative fat mass, body mass index, and waist circumference with diabetes-related mortality by quintiles – NHANES 1999-2018. 23
Supplementary Figure S7. Hazard of diabetes-related mortality among participants without chronic disease (heart disease, stroke, cancer, and renal failure) at baseline in NHANES 1999-2018. 24
Supplementary Figure S8. Hazard of diabetes-related mortality among participants with a follow-up of at least 3 years in NHANES 1999-2018. 25
Supplementary Figure S9. Associations of relative fat mass, body mass index, and waist circumference with diabetes-related mortality among never smokers in NHANES 1999-2018. 26
Supplementary Figure S10. Hazard of diabetes-related mortality according to RFM (relative fat mass) by smoking status in men. 27
Supplementary Figure S11. Hazard of diabetes-related mortality according to waist circumference values, stratified by age categories in women. 28
Supplementary Figure S12. Associations of relative fat mass, body mass index, and waist circumference with heart disease mortality in NHANES 1999-2018. 29
Supplementary Figure S13. Associations of relative fat mass, body mass index, and waist circumference with all-cause mortality in NHANES 1999-2018. 30
Supplementary Figure S14. Hazard of diabetes-related mortality in NHANES III (1988-1994) according to anthropometric indexes. 31
Supplementary Figure S15. Hazard of diabetes-related mortality among participants with a follow-up of at least 3 years in NHANES 1988-1994. 32
Supplementary Figure S16. Associations of relative fat mass, body mass index, and waist circumference with diabetes-related mortality among participants without heart disease, stroke, cancer, and renal failure at baseline in NHANES III (1988-1994). 33
Supplementary Figure S17. Associations of relative fat mass, body mass index, and waist circumference with heart disease mortality in NHANES III (1988-1994). 34
Supplementary Figure S18. Associations of relative fat mass, body mass index, and waist circumference with all-cause mortality in NHANES III (1988-1994). 35
References 36

Supplementary Methods

Data sources

NHANES is a series of cross-sectional two-year survey cycles conducted in multistage national area probability samples of the non-institutionalized population of the United States to assess their health and nutritional status.[1] The analysis performed in the present study included a subset of NHANES data, from 1999-2000, which is the first survey cycle of the last series, through 2017-2018, which is the latest survey cycle for which a follow-up of the vital status of the participants is available (until 2019). Data collected during the survey cycles from 1999-2000 through 2017-2018 were merged following the recommended guidelines.[2]

Definition of covariates and other variables

Education level, smoking status, and ethnicity were self-reported. Education level was categorized as less than 9th grade; 9th to 11th grade; and 12th grade or higher. Smoking status was categorized as current smoker (if smoked at least 100 cigarettes in life and currently smokes) or never/former smoker (never smoker if never smoked or smoked less than 100 cigarettes in life; former smoker if smoked at least 100 cigarettes in life but currently does not smoke).[3] Binary categorization of smoking status was chosen to ensure an adequate number of events per subcategory to obtain reliable estimates and because, in the adjusted model, former smokers and never smokers had similar lower risk of diabetes-related mortality compared with current smokers. Ethnicity was classified as Mexican American, European American, African American, or other ethnicity.

Renal failure was defined as an estimated glomerular filtration rate less than 15 mL/min/1.73 m^2 or self-reported based on serum creatinine, age, and ethnicity.[4] Serum creatinine was standardized as previously reported.[5]

Diabetes was defined as glycated hemoglobin equal to or higher than 6.5% or self-reported.

Model assumptions and model fit

The assumption of proportional hazards over the follow-up period was assessed by visual examination of Schoenfeld residuals.[6] The linearity assumption of continuous variables was visually examined using Martingale residuals.[7] When the assumption of linearity was not met, variables were modelled using restricted cubic splines. The number of knots was chosen to identify the best model fit based on the Akaike information criterion (AIC).[8] Lower AIC values indicate better fit.[9] The location of knots was based on recommended quantiles,[10] depending on the number of knots chosen (Supplementary Table S1). Model assumptions were assessed using the “survminer” package (R version 4.3.1; The R Foundation for Statistical Computing, Vienna, Austria, www.R-project.org).[11] Nonlinearity was tested using the likelihood ratio test.[12]

Supplementary Table S1. Number of knots used for cubic spline regression models.*					
	**NHANES 1999-2018**			**NHANES 1988-1994**	
	**Women**	**Men**		**Women**	**Men**
Diabetes-related mortality	3	3		3	3
Heart disease mortality	5	5		5	4
All-cause mortality	4	3		3	3

* The number of knots was consistent across models comparing anthropometric indexes within each cause of mortality The place of knots was selected based on Harrell's recommendations [10] as follows: for 3 knots, at 10th, 50th, and 90th percentiles; for 4 knots, at 5th, 35th, 65th , and 95th percentiles; and for 5 knots, at 5th, 27.5^{th}, 50th, 72.5^{th}, and 95th percentiles.

Supplementary Table S2. Associations of relative fat mass, body mass index, and waist circumference with diabetes-related mortality, by quintiles. NHANES 1999-2018.*

**Women (23,355)**			
	**RFM †**	**BMI ‡**	**WC**
	**Hazard ratio (95% CI)**	**Hazard ratio (95% CI)**	**Hazard ratio (95% CI)**
Q1	1.00 (Reference)	1.00 (Reference)	1.00 (Reference)
Q2	2.04 (0.87-4.81)	2.21 (1.26-3.90)	1.74 (0.83-3.64)
Q3	2.08 (1.01-4.29)	1.86 (1.09-3.18)	1.98 (1.01-3.86)
Q4	2.69 (1.34-5.39)	2.02 (1.21-3.39)	2.05 (1.07-3.92)
Q5	5.72 (2.74-11.94)	5.23 (2.91-9.41)	5.42 (2.79-10.52)
**Men (23,181)**			
Q1	1.00 (Reference)	1.00 (Reference)	1.00 (Reference)
Q2	0.91 (0.49-1.70)	0.79 (0.52-1.18)	0.76 (0.42-1.37)
Q3	1.10 (0.57-2.11)	0.87 (0.60-1.24)	0.94 (0.55-1.61)
Q4	1.28 (0.73-2.26)	1.25 (0.85-1.84)	1.28 (0.76-2.16)
Q5	3.54 (1.97-6.36)	2.59 (1.74-3.85)	2.91 (1.74-4.85)

* Estimates represent weighted adjusted hazard ratios obtained from Cox regression models for RFM, BMI, and WC. Hazard ratios were adjusted for age, ethnicity, education level, and smoking status. For comparison purposes among indexes, the first quintile (Q1) of each index was arbitrarily chosen as the reference.
† RFM was calculated as follows: 64 − (20 × height/waist circumference) + (12 × sex); sex equals 0 for men and 1 for women; height and waist circumference are measured in the same units.
‡ BMI was calculated as body weight in kilograms divided by the square of the height in meters.
BMI, body mass index; CI, confidence interval; RFM, relative fat mass; WC, waist circumference.

Supplementary Table S3. Associations of relative fat mass, body mass index, and waist circumference with diabetes-related mortality, by quintiles, after further adjustment for muscle mass percentage. NHANES 1999-2006.*

	RFM †		BMI ‡		WC	
	Model 1	Model 2	Model 1	Model 2	Model 1	Model 2
	Hazard ratio (95% CI)	Hazard ratio (95% CI)	Hazard ratio (95% CI)	Hazard ratio (95% CI)	Hazard ratio (95% CI)	Hazard ratio (95% CI)
**Women (7,490)**						
Q1	1.00 (Reference)	1.00 (Reference)	1.00 (Reference)	1.00 (Reference)	1.00 (Reference)	1.00 (Reference)
Q2	3.52 (0.78-15.89)	4.70 (1.00-22.07)	2.73 (1.09-6.81)	4.02 (1.58-10.24)	3.76 (0.94-15.04)	4.90 (1.20-20.00)
Q3	3.36 (0.77-14.67)	5.48 (1.13-26.58)	2.72 (1.20-6.18)	4.96 (1.78-13.82)	4.15 (1.05-16.49)	6.25 (1.43-27.32)
Q4	5.36 (1.39-20.64)	9.95 (1.98-50.00)	2.25 (1.04-4.83)	4.89 (1.85-12.94)	3.76 (1.12-12.66)	6.59 (1.57-27.58)
Q5	8.70 (2.04-37.07)	20.10 (3.91-103.34)	6.14 (2.50-15.08)	17.79 (5.44-58.12)	9.03 (2.31-35.29)	19.25 (4.29-86.38)
**Men (7,691)**						
Q1	1.00 (Reference)	1.00 (Reference)	1.00 (Reference)	1.00 (Reference)	1.00 (Reference)	1.00 (Reference)
Q2	0.80 (0.28-2.23)	0.73 (0.25-2.15)	0.75 (0.44-1.30)	0.63 (0.36-1.10)	0.43 (0.18-1.03)	0.36 (0.15-0.87)
Q3	1.07 (0.42-2.70)	0.93 (0.34-2.49)	0.93 (0.53-1.63)	0.67 (0.38-1.20)	0.80 (0.37-1.74)	0.61 (0.25-1.48)
Q4	1.32 (0.61-2.85)	1.09 (0.44-2.73)	1.30 (0.73-2.33)	0.86 (0.47-1.56)	1.12 (0.55-2.29)	0.76 (0.33-1.76)
Q5	3.58 (1.62-7.91)	2.70 (1.02-7.20)	2.66 (1.52-4.63)	1.34 (0.69-2.62)	2.51 (1.22-5.16)	1.42 (0.57-3.55)

* Estimates represent weighted adjusted hazard ratios obtained from Cox regression models for RFM, BMI, and WC.
Model 1 was adjusted for age, ethnicity, education level, and smoking status.
Model 2 was further adjusted for lean body mass (excluding bone mass) as a surrogate for muscle mass.
RFM, BMI, WC, age, and lean body mass were modelled as continuous variables.
For comparison purposes among indexes, the first quintile (Q1) of each index was arbitrarily chosen as the reference.
† RFM was calculated as follows: 64 − (20 × height/waist circumference) + (12 × sex); sex equals 0 for men and 1 for women; height and waist circumference are measured in the same units.
‡ BMI was calculated as body weight in kilograms divided by the square of the height in meters.
BMI, body mass index; CI, confidence interval; RFM, relative fat mass; WC, waist circumference.

Supplementary Table S4. Competing risk regression analysis for diabetes mortality where diabetes was the underlying cause of death. NHANES 1999-2014.

	**Women**				**Men**		
	**No competing risk**	**Competing risk***	**Multiple competing risk †**		**No competing risk**	**Competing risk***	**Multiple competing risk †**
	**Adjusted hazard ratio (95% CI)**	**Adjusted subhazard ratio (95% CI)**	**Adjusted subhazard ratio (95% CI)**		**Adjusted hazard ratio (95% CI)**	**Adjusted subhazard ratio (95% CI)**	**Adjusted subhazard ratio (95% CI)**
RFM	1.17 (1.08-1.26)	1.16 (1.09-1.24)	1.16 (1.09-1.24)		1.09 (1.01-1.18)	1.09 (1.01-1.17)	1.08 (1.01-1.16)
BMI	1.08 (1.05-1.12)	1.08 (1.05-1.11)	1.08 (1.05-1.11)		1.04 (0.99-1.10)	1.04 (0.99-1.09)	1.04 (0.99-1.09
WC	1.05 (1.03-1.07)	1.05 (1.03-1.06)	1.05 (1.03-1.06)		1.03 (1.00-1.05)	1.02 (1.00-1.05)	1.02 (1.00-1.04)

* Competing risk analysis with heart disease as the competing event.
† Competing risk with heart disease, cancer, chronic lower respiratory diseases, accidents (unintentional injuries), cerebrovascular diseases, Alzheimer's disease, influenza and pneumonia, kidney, and all other causes, as the competing events.
Hazard ratios and subhazard ratios (for competing risk) were adjusted for age, ethnicity, education level, and smoking status. RFM, BMI, WC, and age were modelled as continuous variables.
BMI, body mass index; CI, confidence interval; RFM, relative fat mass; WC, waist circumference.
Although the information on diabetes as a contributing cause was available for the entire study period (1999-2018), the information on cause-specific mortality by 10 categories was only available for the period 1999-2014. From 2015 to 2018, only information for heart disease and cancer as the underlying cause of death was available. Therefore, the competing risk analysis was limited to the period 1999-2014.
Hazard ratio should be interpreted as per 1 unit increment of the corresponding anthropometric index.

Supplementary Table S5. Discriminatory prediction of diabetes-related mortality among adult participants of NHANES 1999-2018.*				
	**Women**		**Men**	
	**C-index (95% CI) †**	**P value (vs. RFM) ‡**	**C-index (95% CI) †**	**P value (vs. RFM) ‡**
**All participants**				
n	23,354		23,181	
Diabetes deaths, n	319		424	
RFM	0.73 (0.69-0.77)		0.71 (0.67-0.75)	
BMI	0.63 (0.58-0.68)	P<0.001	0.59 (0.55-0.64)	P<0.001
WC	0.71 (0.67-0.75)	P<0.001	0.67 (0.63-0.71)	P<0.001
**Excluding heart disease, stroke, cancer, and renal failure at baseline**				
n	19,534		19,000	
Diabetes deaths, n	171		231	
RFM	0.73 (0.67-0.80)		0.74 (0.68-0.79)	
BMI	0.64 (0.57-0.72)	P<0.001	0.63 (0.57-0.68)	P<0.001
WC	0.71 (0.65-0.78)	P=0.006	0.69 (0.64-0.75)	P<0.001
**Excluding the first 3 years of follow-up**				
n	20,534		20,190	
Diabetes deaths, n	256		346	
RFM	0.73 (0.68-0.77)		0.73 (0.70-0.77)	
BMI	0.64 (0.58-0.69)	P<0.001	0.63 (0.59-0.67)	P<0.001
WC	0.71 (0.66-0.75)	P=0.0002	0.70 (0.66-0.74)	P<0.001
**Excluding diabetes at baseline**				
n	20,008		19,624	
Diabetes deaths, n	69		106	
RFM	0.61 (0.52-0.70)		0.71 (0.64-0.78)	
BMI	0.51 (0.41-0.61)	P<0.001	0.59 (0.5-0.67)	P<0.001
WC	0.56 (0.47-0.65)	P<0.001	0.68 (0.59-0.76)	P<0.001
**Never-smokers**				
n	14,800		10,290	
Diabetes deaths, n	189		145	
RFM	0.74 (0.70-0.79)		0.72 (0.66-0.78)	
BMI	0.65 (0.59-0.70)	P<0.001	0.59 (0.52-0.66)	P<0.001
WC	0.72 (0.67-0.76)	P=0.0002	0.67 (0.60-0.75)	P=0.0004

* Sample size (n) represents unweighted data. Estimates represent weighted data.
† C-index estimates are unadjusted.
‡ P values indicate how incompatible the observed data may be with the null hypothesis that RFM's discriminatory prediction is not superior to either BMI or WC. P values were obtained using the Wald test with Bonferroni correction
BMI, body mass index; RFM, relative fat mass; WC, waist circumference.

Supplementary Table S6. Discriminatory prediction of heart disease mortality among adult participants of NHANES 1999-2018.*

	Women		Men	
	**C-index (95% CI) †**	**P value (vs. RFM) ‡**	**C-index (95% CI) †**	**P value (vs. RFM) ‡**
**All participants**				
n	23,354		23,181	
Heart disease deaths, n	596		918	
RFM	0.64 (0.61-0.66)		0.67 (0.64-0.69)	
BMI	0.53 (0.50-0.56)	P<0.001	0.56 (0.53-0.58)	P<0.001
WC	0.60 (0.57-0.63)	P<0.001	0.64 (0.61-0.66)	P<0.001
**Excluding heart disease, stroke, cancer, and renal failure at baseline**				
n	19,534		19,000	
Heart disease deaths, n	337		443	
RFM	0.64 (0.60-0.67)		0.66 (0.61-0.70)	
BMI	0.54 (0.50-0.57)	P<0.001	0.57 (0.52-0.61)	P<0.001
WC	0.60 (0.56-0.63)	P<0.001	0.64 (0.60-0.68)	P=0.001
**Excluding the first 3 years of follow-up**				
n	20,534		20,190	
Heart disease deaths, n	496		733	
RFM	0.64 (0.62-0.67)		0.68 (0.66-0.71)	
BMI	0.54 (0.51-0.58)	P<0.001	0.58 (0.55-0.60)	P<0.001
WC	0.61 (0.58-0.64)	P<0.001	0.65 (0.63-0.68)	P<0.001
**Excluding diabetes at baseline**				
n	20,008		19,624	
Heart disease deaths, n	389		622	
RFM	0.61 (0.58-0.64)		0.65 (0.62-0.69)	
BMI	0.51 (0.48-0.54)	P<0.001	0.53 (0.50-0.57)	P<0.001
WC	0.57 (0.54-0.60)	P<0.001	0.62 (0.59-0.65)	P<0.001
**Never-smokers**				
n	14,800		10,290	
Heart disease deaths, n	329		282	
RFM	0.65 (0.62-0.69)		0.67 (0.63-0.72)	
BMI	0.54 (0.50-0.58)	P<0.001	0.56 (0.51-0.60)	P<0.001
WC	0.61 (0.57-0.64)	P<0.001	0.63 (0.58-0.67)	P<0.001

* Sample size (n) represents unweighted data. Estimates represent weighted data.
† C-index estimates are unadjusted.
‡ P values indicate how incompatible the observed data may be with the null hypothesis that RFM's discriminatory prediction is not superior to either BMI or WC. P values were obtained using the Wald test with Bonferroni correction
BMI, body mass index; RFM, relative fat mass; WC, waist circumference.

**Supplementary Table S7. Discriminatory prediction of all-cause mortality among adult participants of NHANES 1999-2018.***				
	**Women**		**Men**	
	**C-index (95% CI) †**	**P value (vs. RFM) ‡**	**C-index (95% CI) †**	**P value (vs. RFM) ‡**
**All participants**				
n	23,354		23,181	
Deaths, n	2,551		3,550	
RFM	0.60 (0.59-0.62)		0.61 (0.60-0.63)	
BMI	0.51 (0.50-0.53)	P<0.001	0.51 (0.50-0.53)	P<0.001
WC	0.58 (0.56-0.59)	P<0.001	0.59 (0.58-0.60)	P<0.001
**Excluding heart disease, stroke, cancer, and renal failure at baseline**				
n	19,534		19,000	
Deaths, n	1,505		1,928	
RFM	0.61 (0.59-0.62)		0.60 (0.58-0.62)	
BMI	0.52 (0.50-0.54)	P<0.001	0.51 (0.49-0.53)	P<0.001
WC	0.58 (0.56-0.60)	P<0.001	0.58 (0.55-0.60)	P<0.001
**Excluding the first 3 years of follow-up**				
n	20,534		20,190	
Heart disease deaths, n	2,098		2,815	
RFM	0.60 (0.59-0.62)		0.63 (0.62-0.64)	
BMI	0.51 (0.50-0.53)	P<0.001	0.53 (0.51-0.54)	P<0.001
WC	0.58 (0.56-0.59)	P<0.001	0.61 (0.59-0.62)	P<0.001
**Excluding diabetes at baseline**				
n	20,008		19,624	
Deaths, n	1,789		2,528	
RFM	0.58 (0.56-0.59)		0.59 (0.58-0.61)	
BMI	0.52 (0.50-0.53)	P=0.003	0.51 (0.49-0.53)	P<0.001
WC	0.55 (0.53-0.56)	P<0.001	0.57 (0.55-0.59)	P<0.001
**Never-smokers**				
n	14,800		10,290	
Deaths, n	1,288		995	
RFM	0.62 (0.60-0.65)		0.62 (0.60-0.65)	
BMI	0.52 (0.50-0.55)	P<0.001	0.51 (0.48-0.54)	P<0.001
WC	0.59 (0.56-0.61)	P<0.001	0.58 (0.55-0.60)	P<0.001

* Sample size (n) represents unweighted data. Estimates represent weighted data.
† C-index estimates are unadjusted.
‡ P values indicate how incompatible the observed data may be with the null hypothesis that RFM’s discriminatory prediction is not superior to either BMI or WC. P values were obtained using the Wald test with Bonferroni correction
BMI, body mass index; RFM, relative fat mass; WC, waist circumference.

Supplementary Table S8. Discriminatory prediction of RFM, DXA-measured whole body fat, and intra-abdominal fat for mortality among adult participants of NHANES 2005-2006.*

	Women		Men	
	**C-index (95% CI) †**	**P value (RFM vs. Intra-abdominal fat) ‡**	**C-index (95% CI) †**	**P value (RFM vs. Intra-abdominal fat) ‡**
**Diabetes-related mortality**				
n	1,437		1,532	
Diabetes deaths, n	16		17	
RFM	0.76 (0.68-0.85)		0.91 (0.85-0.98)	
DXA-whole body fat %	0.68 (0.54-0.82)		0.87 (0.77-0.96)	
DXA-android fat %	0.66 (0.57-0.76)	0.0003	0.85 (0.75-0.94)	0.12
**Heart disease mortality**				
n	1,437		1,532	
Heart disease deaths, n	26		36	
RFM	0.68 (0.57-0.79)		0.68 (0.57-0.80)	
DXA-whole body fat %	0.65 (0.52-0.78)		0.62 (0.52-0.72)	
DXA-android fat %	0.61 (0.50-0.73)	0.50	0.58 (0.46-0.70)	0.046
**All-cause mortality**				
n	1,437		1,532	
Deaths, n	113		149	
RFM	0.60 (0.53-0.67)		0.62 (0.54-0.71)	
DXA-whole body fat %	0.55 (0.48-0.62)		0.58 (0.52-0.65)	
DXA-android fat %	0.54 (0.48-0.60)	0.016	0.54 (0.47-0.62)	0.009

* Sample size (n) represents unweighted data. Estimates represent weighted data.
† C-index estimates are unadjusted.
‡ P values indicate how incompatible the observed data may be with the null hypothesis (that RFM's discriminatory prediction is not superior to DXA-android fat).
CI, confidence interval; DXA, dual-energy X-ray absorptiometry; RFM, relative fat mass.

Supplementary Table S9. Characteristics of adult participants of NHANES III (1988-1994), overall and among those who died from diabetes as the underlying or contributing cause of death.*

Characteristic	All	Women		Men	
		All	Diabetes deaths	All	Diabetes deaths
n	14448	7,536	366	6,912	361
Mean age (SD), years	43.7 (16.0)	44.5 (16.4)	60.1 (14.7)	42.8 (15.5)	55.9 (16.2)
Ethnicity, n (%)					
Mexican American	4,038 (5.1)	1,962 (4.6)	111 (4.7)	2,076 (5.6)	124 (6.0)
European American	5,669 (76.3)	3,005 (75.9)	133 (74.3)	2,664 (76.6)	132 (79.1)
African American	4,145 (10.8)	2,231 (11.5)	111 (15.3)	1,914 (10.0)	97 (11.9)
Other ethnicity	596 (7.9)	338 (8.0)	11 (5.7)	258 (7.8)	8 (3.1)
Mean body weight (SD), kg	75.8 (18.0)	69.6 (17.3)	81.6 (25.9)	82.3 (16.3)	90.9 (25.8)
Mean height (SD), cm	168.7 (9.8)	162.0 (6.9)	160.4 (8.3)	175.7 (7.2)	174.8 (7.9)
Mean WC (SD), cm	91.8 (14.7)	88.6 (15.4)	104.3 (18.7)	95.3 (13.0)	105.7 (17.7)
Mean BMI (SD), kg/m^2 †	26.5 (5.6)	26.5 (6.4)	31.6 (9.3)	26.6 (4.7)	29.6 (7.0)
Mean RFM (SD), % ‡	32.6 (8.2)	38.4 (6.4)	44.6 (5.9)	26.5 (4.9)	30.3 (5.5)
Current smoker, n (%)					
No	10,466 (70.7)	5,805 (74.1)	282 (71.1)	4,661 (67.3)	255 (69.4)
Yes	3,982 (29.3)	1,731 (26.0)	84 (28.9)	2,251 (32.7)	106 (30.6)
Education, n (%)					
Less than 9th grade	3,244 (10.6)	1,554 (10.4)	141 (22.2)	1,690 (10.9)	136 (20.9)
9th to 11th grade	2,387 (13.3)	1,216 (12.6)	84 (22.5)	1,171 (14.0)	64 (20.3)
12th grade or higher	8,817 (76.1)	4,766 (77.1)	141 (55.3)	4,051 (75.1)	161 (58.8)
Median follow-up (IQR), years	27.1 (23.0-28.8)	27.1 (23.7-28.9)	15.6 (9.4-20.5)	27.1 (22.4-28.8)	18.3 (10.5-23.2)

* Sample size (n) represents unweighted data. Estimates represent weighted data. Percentages may not total 100 due to rounding.
† BMI was calculated as body weight in kilograms divided by the square of the height in meters.
‡ RFM was calculated as follows: 64 − (20 × height/waist circumference) + (12 × sex); sex equals 0 for men and 1 for women; height and waist circumference are measured in the same units.
BMI, body mass index; IQR, interquartile range; RFM, relative fat mass; SD, standard deviation; WC, waist circumference.

Supplementary Table S10. Associations of relative fat mass, body mass index and waist circumference with diabetes-related mortality among adult participants of NHANES III (1988-1994).*

	RFM†, %	Hazard ratio (95% CI)	BMI‡, kg/m^2	Hazard ratio (95% CI)	WC, cm	Hazard ratio (95% CI)
**Women (n = 7,536)**						
	25	0.37 (0.06-2.11)	11	0.31 (0.11-0.88)	51	0.13 (0.02-0.80)
	28	0.47 (0.13-1.69)	14	0.44 (0.21-0.91)	59	0.22 (0.06-0.84)
	31	0.59 (0.26-1.36)	17	0.63 (0.41-0.95)	67	0.36 (0.15-0.88)
	34	0.75 (0.51-1.11)	20	0.89 (0.81-0.98)	75	0.61 (0.40-0.93)
25th Percentile	37	1.00 (Reference)	23	1.00 (Reference)	83	1.00 (Reference)
	40	1.46 (1.12-1.90)	26	1.74 (1.22-2.49)	91	1.62 (1.20-2.18)
	43	2.43 (1.68-3.50)	29	2.36 (1.54-3.62)	99	2.55 (1.67-3.89)
	46	4.45 (3.01-6.57)	32	3.15 (2.03-4.88)	107	3.93 (2.53-6.09)
	49	8.42 (5.42-13.07)	35	4.18 (2.72-6.43)	115	6.02 (3.89-9.30)
	52	15.96 (9.33-27.30)	38	5.55 (3.59-8.57)	123	9.21 (5.82-14.55)
**Men (n = 6,912)**						
	13	0.79 (0.23-2.78)	13	0.31 (0.09-1.15)	58	0.51 (0.12-2.13)
	16	0.82 (0.32-2.07)	16	0.42 (0.16-1.10)	66	0.60 (0.21-1.73)
	19	0.85 (0.47-1.54)	19	0.56 (0.30-1.05)	74	0.70 (0.35-1.41)
	22	0.89 (0.68-1.16)	22	0.75 (0.56-1.01)	82	0.83 (0.60-1.15)
25th Percentile	25	1.00 (Reference)	25	1.00 (Reference)	90	1.00 (Reference)
	28	1.34 (1.14-1.56)	28	1.32 (1.13-1.55)	98	1.29 (1.07-1.56)
	31	2.18 (1.73-2.74)	31	1.74 (1.41-2.15)	106	1.80 (1.41-2.30)
	34	3.89 (2.79-5.44)	34	2.28 (1.77-2.95)	114	2.61 (1.96-3.46)
	37	7.02 (4.37-11.27)	37	3.00 (2.15-4.18)	122	3.78 (2.65-5.40)
	40	12.65 (6.76-23.66)	40	3.93 (2.57-6.01)	130	5.49 (3.51-8.59)

* Estimates represent adjusted hazard ratios obtained from Cox regression models for RFM, BMI, and WC modelled using restricted cubic splines. Hazard ratios were adjusted for age (as restricted cubic splines), ethnicity, education level, and smoking status. The rounded weighted 25th percentile of each index was arbitrarily chosen as the reference. Index values are shown in increments of approximately 0.5 of their standard deviation to facilitate comparison.
† RFM was calculated as follows: 64 − (20 × height/waist circumference) + (12 × sex); sex equals 0 for men and 1 for women; height and waist circumference were measured in the same units.
‡ BMI was calculated as body weight in kilograms divided by the square of the height in meters.
BMI, body mass index; CI, confidence interval; RFM, relative fat mass; WC, waist circumference.

Supplementary Table S11. Associations of relative fat mass, body mass index, and waist circumference with heart disease mortality among adult participants of NHANES III (1988-1994).*

	RFM†, %	Hazard ratio (95% CI)	BMI‡, kg/m^2	Hazard ratio (95% CI)	WC, cm	Hazard ratio (95% CI)
**Women (7,536)**						
	25	1.00 (0.54-1.83)	11	2.12 (0.41-11.07)	51	0.92 (0.46-1.83)
	28	0.98 (0.63-1.54)	14	1.73 (0.52-5.75)	59	0.93 (0.56-1.56)
	31	0.97 (0.73-1.30)	17	1.41 (0.66-2.99)	67	0.95 (0.68-1.33)
	34	0.97 (0.85-1.11)	20	1.15 (0.84-1.56)	75	0.97 (0.82-1.14)
25th Percentile	37	1.00 (Reference)	23	1.00 (Reference)	83	1.00 (Reference)
	40	1.11 (1.02-1.21)	26	1.09 (0.85-1.39)	91	1.10 (0.99-1.23)
	43	1.38 (1.21-1.57)	29	1.37 (1.02-1.84)	99	1.33 (1.14-1.55)
	46	1.84 (1.52-2.23)	32	1.74 (1.32-2.30)	107	1.70 (1.43-2.03)
	49	2.54 (1.91-3.36)	35	2.08 (1.56-2.77)	115	2.24 (1.80-2.78)
	52	3.49 (2.37-5.15)	38	2.39 (1.79-3.19)	123	2.94 (2.22-3.90)
**Men (6,912)**						
	13	0.93 (0.46-1.86)	13	2.10 (0.76-5.77)	58	1.06 (0.50-2.21)
	16	0.93 (0.56-1.56)	16	1.66 (0.81-3.41)	66	1.04 (0.60-1.79)
	19	0.94 (0.68-1.31)	19	1.32 (0.86-2.01)	74	1.01 (0.71-1.45)
	22	0.95 (0.82-1.10)	22	1.05 (0.91-1.23)	82	0.99 (0.84-1.17)
25th Percentile	25	1.00 (Reference)	25	1.00 (Reference)	90	1.00 (Reference)
	28	1.15 (1.06-1.25)	28	1.24 (1.04-1.48)	98	1.09 (0.99-1.20)
	31	1.47 (1.27-1.70)	31	1.53 (1.17-2.00)	106	1.30 (1.14-1.48)
	34	1.97 (1.55-2.50)	34	1.82 (1.38-2.40)	114	1.61 (1.34-1.93)
	37	2.65 (1.86-3.78)	37	2.13 (1.59-2.86)	122	2.00 (1.55-2.59)
	40	3.56 (2.22-5.73)	40	2.50 (1.77-3.52)	130	2.50 (1.78-3.50)

* Estimates represent adjusted hazard ratios obtained from Cox regression models for RFM, BMI, and WC modelled using restricted cubic splines. Hazard ratios were adjusted for age (as restricted cubic splines), ethnicity, education level, and smoking status. The rounded weighted 25th percentile of each index was arbitrarily chosen as the reference. Index values are shown in increments of approximately 0.5 of their standard deviation to facilitate comparison.
† RFM was calculated as follows: 64 − (20 × height/waist circumference) + (12 × sex); sex equals 0 for men and 1 for women; height and waist circumference were measured in the same units.
‡ BMI was calculated as body weight in kilograms divided by the square of the height in meters.
BMI, body mass index; CI, confidence interval; RFM, relative fat mass; WC, waist circumference.

Supplementary Table S12. Associations of relative fat mass, body mass index, and waist circumference with all-cause mortality among adult participants of NHANES III (1988-1994).*

	RFM†, %	Hazard ratio (95% CI)	BMI‡, kg/m^2	Hazard ratio (95% CI)	WC, cm	Hazard ratio (95% CI)
**Women (7,536)**						
	25	1.05 (0.79-1.40)	11	2.65 (1.23-5.73)	51	0.96 (0.69-1.35)
	28	1.03 (0.83-1.27)	14	2.03 (1.16-3.55)	59	0.97 (0.75-1.25)
	31	1.01 (0.88-1.15)	17	1.56 (1.10-2.21)	67	0.98 (0.83-1.15)
	34	0.99 (0.93-1.05)	20	1.19 (1.04-1.37)	75	0.98 (0.91-1.06)
25th Percentile	37	1.00 (Reference)	23	1.00 (Reference)	83	1.00 (Reference)
	40	1.07 (1.03-1.12)	26	1.09 (0.96-1.25)	91	1.07 (1.02-1.13)
	43	1.25 (1.18-1.34)	29	1.27 (1.09-1.49)	99	1.23 (1.14-1.32)
	46	1.56 (1.41-1.73)	32	1.45 (1.24-1.69)	107	1.48 (1.35-1.61)
	49	1.98 (1.69-2.32)	35	1.63 (1.38-1.92)	115	1.81 (1.61-2.04)
	52	2.51 (2.01-3.14)	38	1.82 (1.55-2.14)	123	2.22 (1.89-2.60)
**Men (6,912)**						
	13	1.11 (0.79-1.57)	13	2.69 (1.49-4.86)	58	1.34 (0.89-2.02)
	16	1.07 (0.83-1.38)	16	2.02 (1.32-3.07)	66	1.24 (0.91-1.68)
	19	1.03 (0.87-1.21)	19	1.51 (1.18-1.94)	74	1.14 (0.94-1.39)
	22	0.99 (0.92-1.07)	22	1.14 (1.05-1.25)	82	1.05 (0.96-1.15)
25th Percentile	25	1.00 (Reference)	25	1.00 (Reference)	90	1.00 (Reference)
	28	1.11 (1.06-1.16)	28	1.11 (1.01-1.23)	98	1.05 (1.00-1.10)
	31	1.36 (1.26-1.48)	31	1.31 (1.13-1.51)	106	1.22 (1.13-1.32)
	34	1.77 (1.53-2.04)	34	1.55 (1.34-1.81)	114	1.50 (1.34-1.68)
	37	2.31 (1.87-2.85)	37	1.85 (1.55-2.20)	122	1.85 (1.58-2.17)
	40	3.01 (2.27-3.98)	40	2.20 (1.77-2.75)	130	2.28 (1.84-2.81)

* Estimates represent adjusted hazard ratios obtained from Cox regression models for RFM, BMI, and WC modelled using restricted cubic splines. Hazard ratios were adjusted for age (as restricted cubic splines), ethnicity, education level, and smoking status. The rounded weighted 25th percentile of each index was arbitrarily chosen as the reference. Index values are shown in increments of approximately 0.5 of their standard deviation to facilitate comparison.
† RFM was calculated as follows: 64 − (20 × height/waist circumference) + (12 × sex); sex equals 0 for men and 1 for women; height and waist circumference were measured in the same units.
‡ BMI was calculated as body weight in kilograms divided by the square of the height in meters.
BMI, body mass index; CI, confidence interval; RFM, relative fat mass; WC, waist circumference.

Supplementary Table S13. Discriminatory prediction of diabetes-related mortality among adult participants of NHANES III (1988-1994).*

	Women		Men	
	**C-index (95% CI) †**	**P value (vs. RFM) ‡**	**C-index (95% CI) †**	**P value (vs. RFM) ‡**
**All participants**				
n	7,536		6,912	
Diabetes deaths, n	366		361	
RFM	0.79 (0.76-0.83)		0.76 (0.72-0.80)	
BMI	0.71 (0.66-0.75)	P<0.001	0.68 (0.63-0.73)	P<0.001
WC	0.78 (0.74-0.82)	P=0.041	0.74 (0.70-0.78)	P=0.002
**Excluding heart disease, stroke, cancer, and renal failure at baseline**				
n	6,360		5,802	
Diabetes deaths, n	260		249	
RFM	0.79 (0.75-0.84)		0.76 (0.71-0.81)	
BMI	0.71 (0.65-0.77)	P<0.001	0.69 (0.63-0.75)	P<0.001
WC	0.78 (0.73-0.83)	P=0.07	0.74 (0.69-0.79)	P=0.019
**Excluding the first 3 years of follow-up**				
n	7,411		6,653	
Diabetes deaths, n	348		330	
RFM	0.79 (0.75-0.83)		0.77 (0.73-0.81)	
BMI	0.71 (0.66-0.76)	P<0.001	0.69 (0.64-0.74)	P<0.001
WC	0.78 (0.74-0.82)	P=0.06	0.75 (0.71-0.79)	P=0.006
**Excluding diabetes at baseline**				
n	6,667		6,215	
Diabetes deaths, n	116		163	
RFM	0.81 (0.75-0.86)		0.82 (0.77-0.86)	
BMI	0.73 (0.67-0.80)	P=0.0002	0.73 (0.68-0.79)	P<0.001
WC	0.80 (0.74-0.86)	P=0.26	0.80 (0.75-0.84)	P=0.002
**Never-smokers**				
n	4,507		2,466	
Diabetes deaths, n	202		82	
RFM	0.81 (0.77-0.85)		0.77 (0.67-0.88)	
BMI	0.72 (0.67-0.77)	P<0.001	0.69 (0.58-0.79)	P=0.008
WC	0.80 (0.76-0.84)	P=0.16	0.75 (0.65-0.86)	P=0.022

* Sample size (n) represents unweighted data. Estimates represent weighted data.
† C-index estimates are unadjusted.
‡ P values indicate how incompatible the observed data may be with the null hypothesis that RFM's discriminatory prediction is not superior to either BMI or WC. P values were obtained using the Wald test with Bonferroni correction.
§ Diagnosed and undiagnosed diabetes identified with measured glycated hemoglobin. Diabetes was defined as a glycated hemoglobin equal to or higher than 6.5% or self-reported.
BMI, body mass index; RFM, relative fat mass; WC, waist circumference.

Supplementary Table S14. Discriminatory prediction of heart disease mortality and all-cause mortality among adult participants of NHANES III (1988-1994).*

	**Women**		**Men**	
	**C-index (95% CI) †**	**P value (vs. RFM) ‡**	**C-index (95% CI) †**	**P value (vs. RFM) ‡**
**Heart disease mortality**				
n	7,536		6,912	
Heart disease deaths, n	751		857	
RFM	0.69 (0.66-0.72)		0.70 (0.67-0.72)	
BMI	0.60 (0.57-0.64)	P<0.001	0.60 (0.57-0.62)	P<0.001
WC	0.67 (0.64-0.70)	P<0.001	0.66 (0.64-0.69)	P<0.001
**All-cause mortality**				
n	7,536		6,912	
Deaths, n	2,702		2,971	
RFM	0.67 (0.65-0.68)		0.67 (0.65-0.68)	
BMI	0.58 (0.57-0.60)	P<0.001	0.57 (0.55-0.59)	P<0.001
WC	0.65 (0.63-0.66)	P<0.001	0.64 (0.62-0.66)	P<0.001

* Sample size (n) represents unweighted data. Estimates represent weighted data.
† C-index estimates are unadjusted.
‡ P values indicate how incompatible the observed data may be with the null hypothesis that RFM's discriminatory prediction is not superior to either BMI or WC. P values were obtained using the Wald test with Bonferroni correction
BMI, body mass index; RFM, relative fat mass; WC, waist circumference.

Supplementary Figure S1. Selection of study participants. Among the fully eligible participants of NHANES 1999-2018, the total proportion of missing data in the variables analyzed was 6.2%: body weight, 1.5% (n=751); height, 1.4% (n=695); waist circumference, 5.5% (n=2,746); smoking status, 0.01% (n=4), and information on vital status, 0.2% (n=110). Among the fully eligible participants of NHANES III, the total proportion of missing data in the variables analyzed was 5.3%: body weight, 0.3% (n=38); height, 0.2% (n=23); waist circumference, 4.6% (n=703); education, 0.6% (n=96); smoking status, 0.01% (n=1), and information on vital status, 0.1% (n=10). Some participants had missing information on more than one variable.

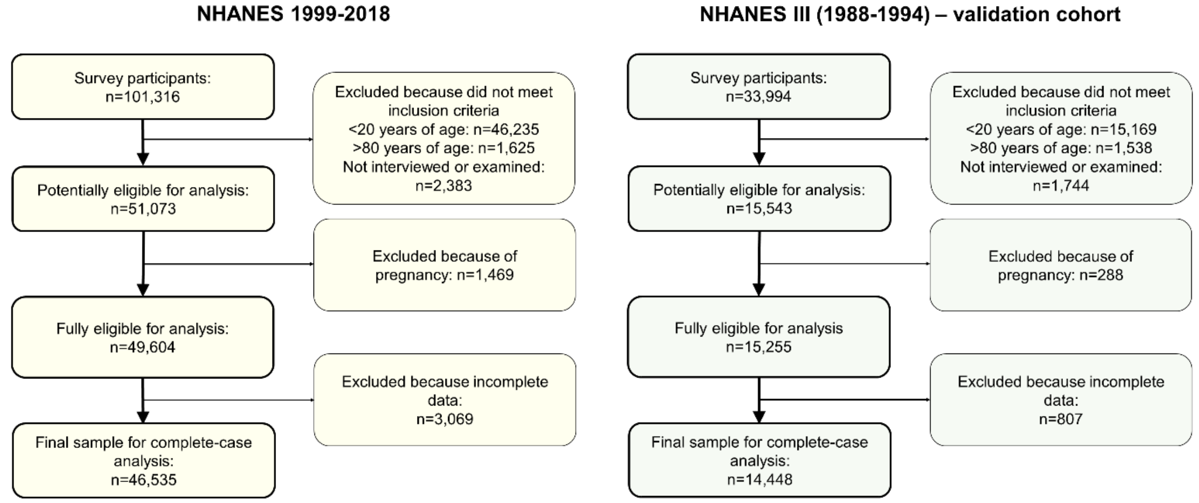

Supplementary Figure S2. Assumption of proportionality – RFM – NHANES 1999-2018. Visual examination of the assumption of proportional hazards of the RFM (relative fat mass) models using Schoenfeld residuals plots.

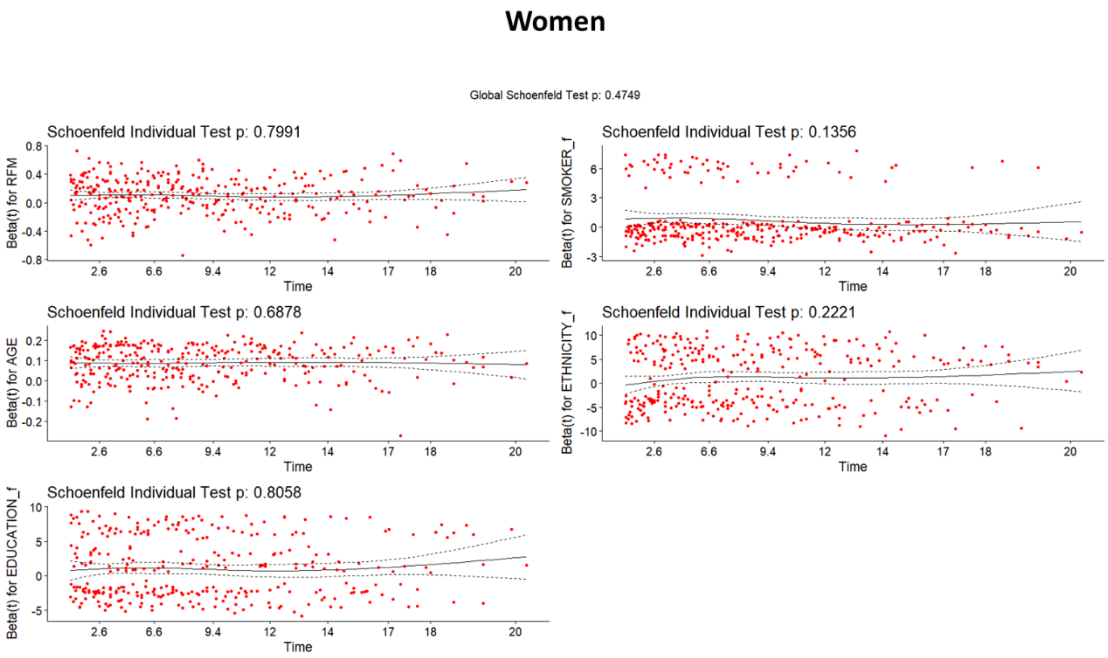

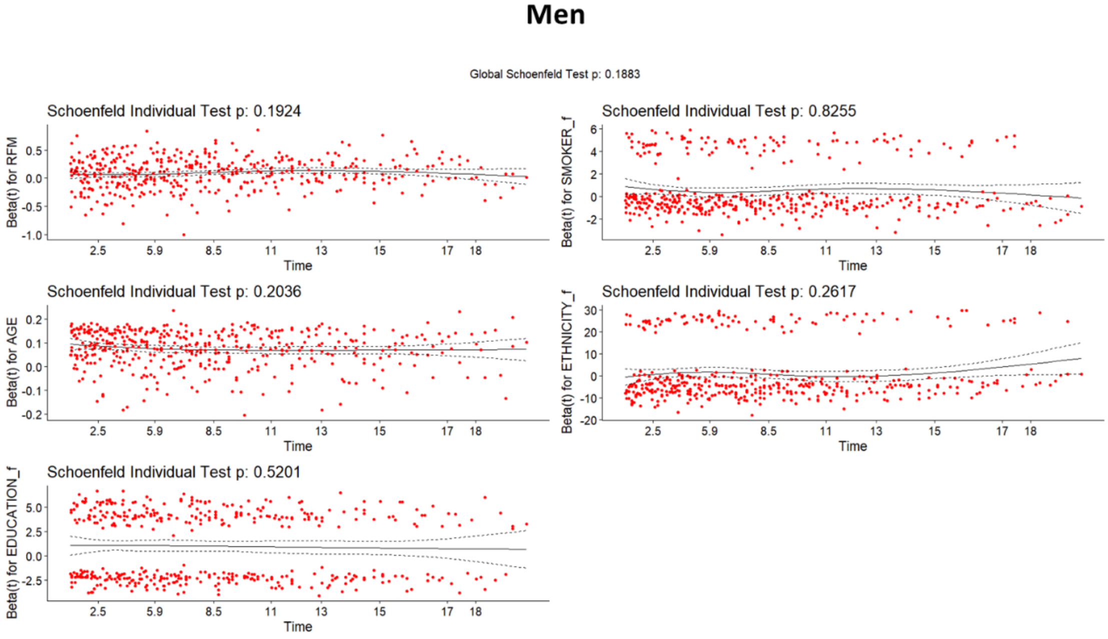

Supplementary Figure S3. Assessment of assumption of proportionality – BMI – NHANES 1999-2018. Visual examination of the assumption of proportional hazards of the BMI (body mass index) models using Schoenfeld residuals plots.

Women

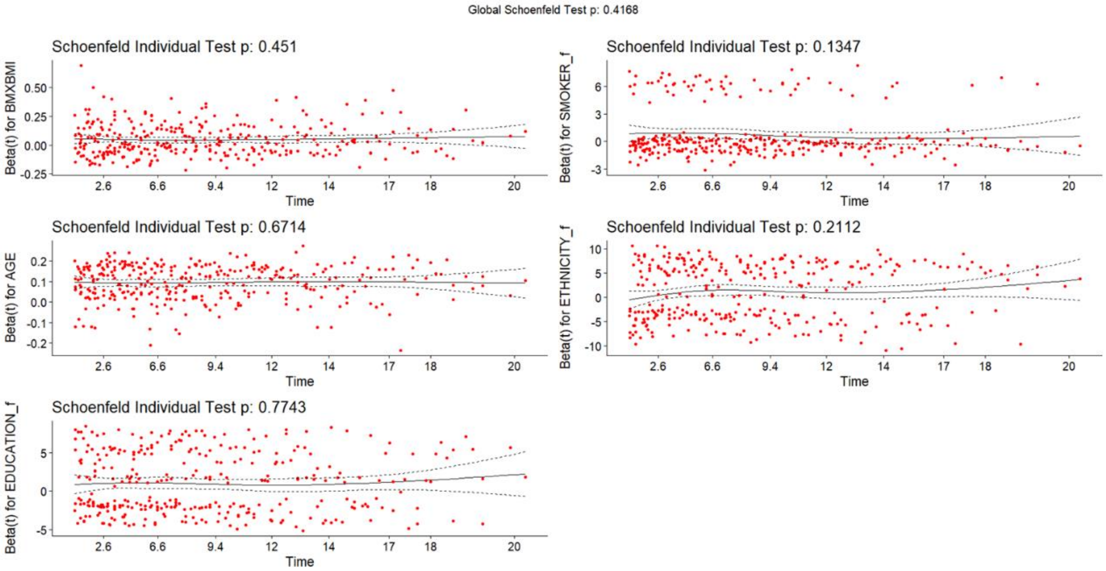

Men

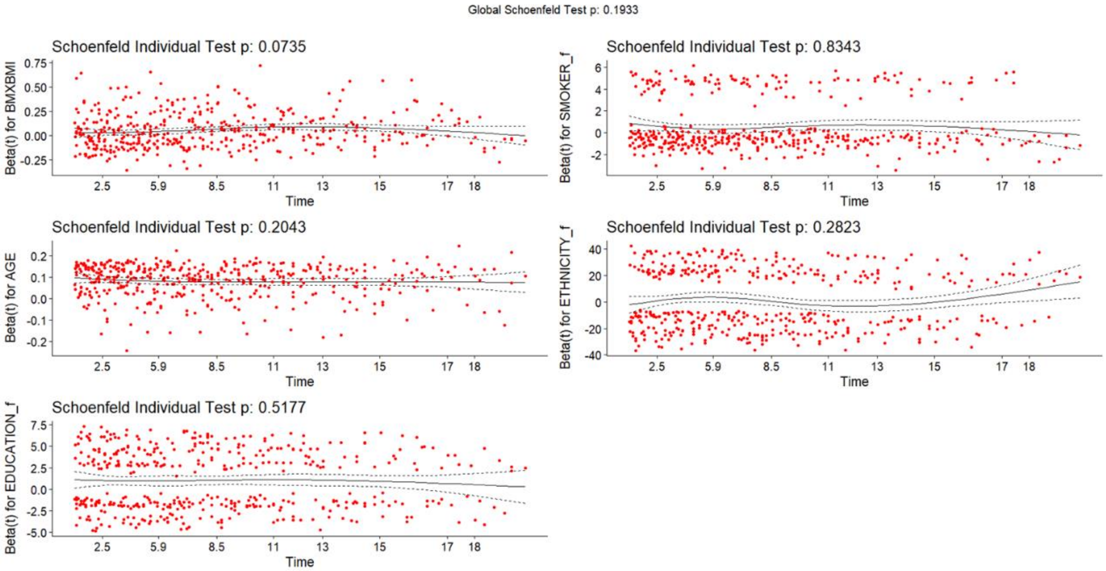

Supplementary Figure S4. Assessment of assumption of proportionality – WC – NHANES 1999-2018. Visual examination of the assumption of proportional hazards of the WC (waist circumference) models using Schoenfeld residuals plots.

Women

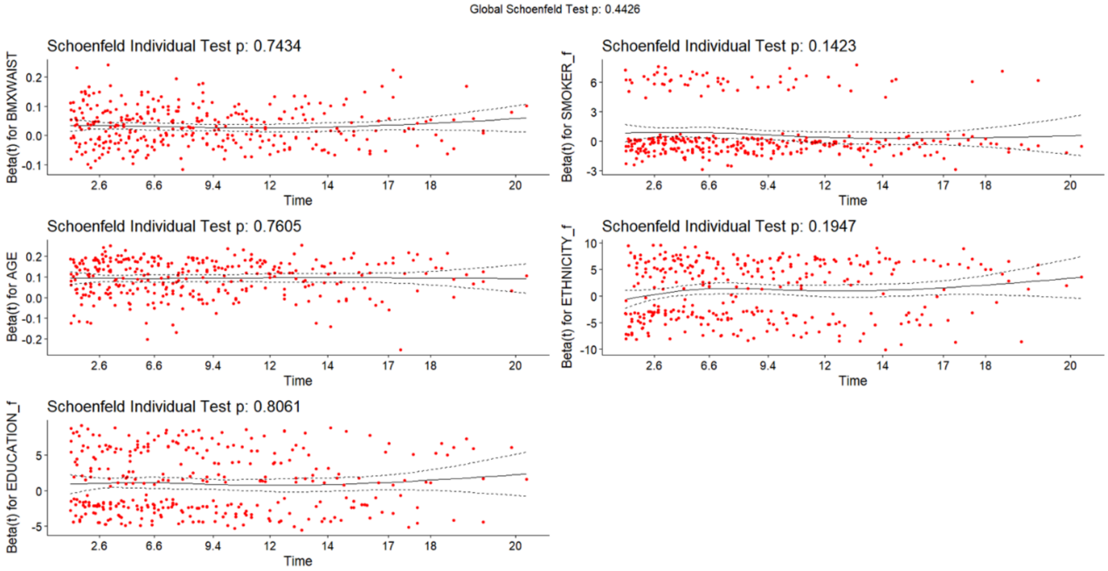

Men

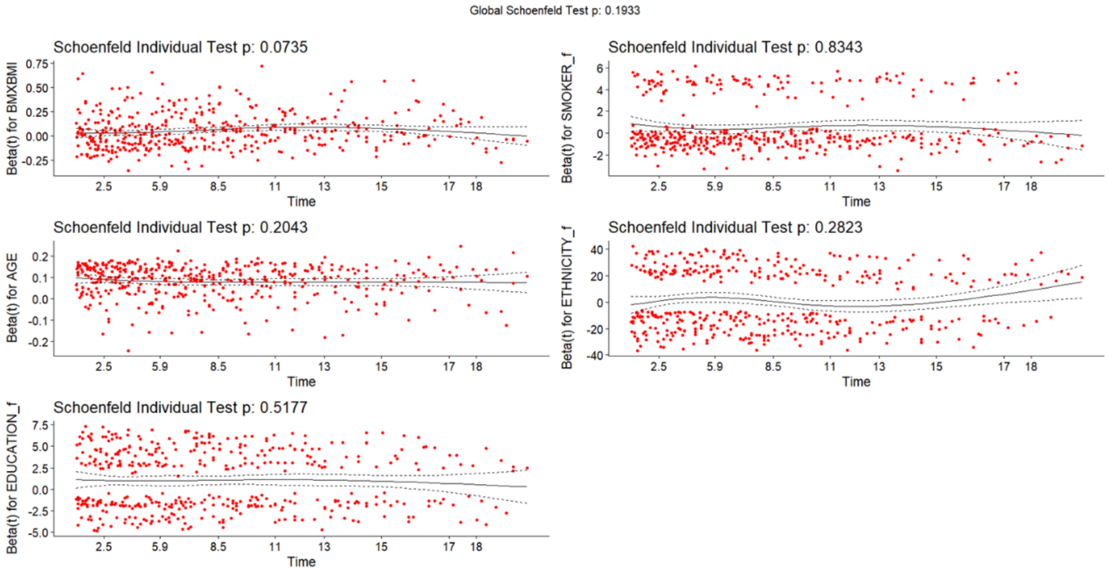

Supplementary Figure S5. Assessment of assumption of linearity – NHANES 1999-2018. Visual examination of the linearity assumption of continuous variables included in the Cox regression models using Martingale residuals plots. RFM, relative fat mass; BMI, body mass index.

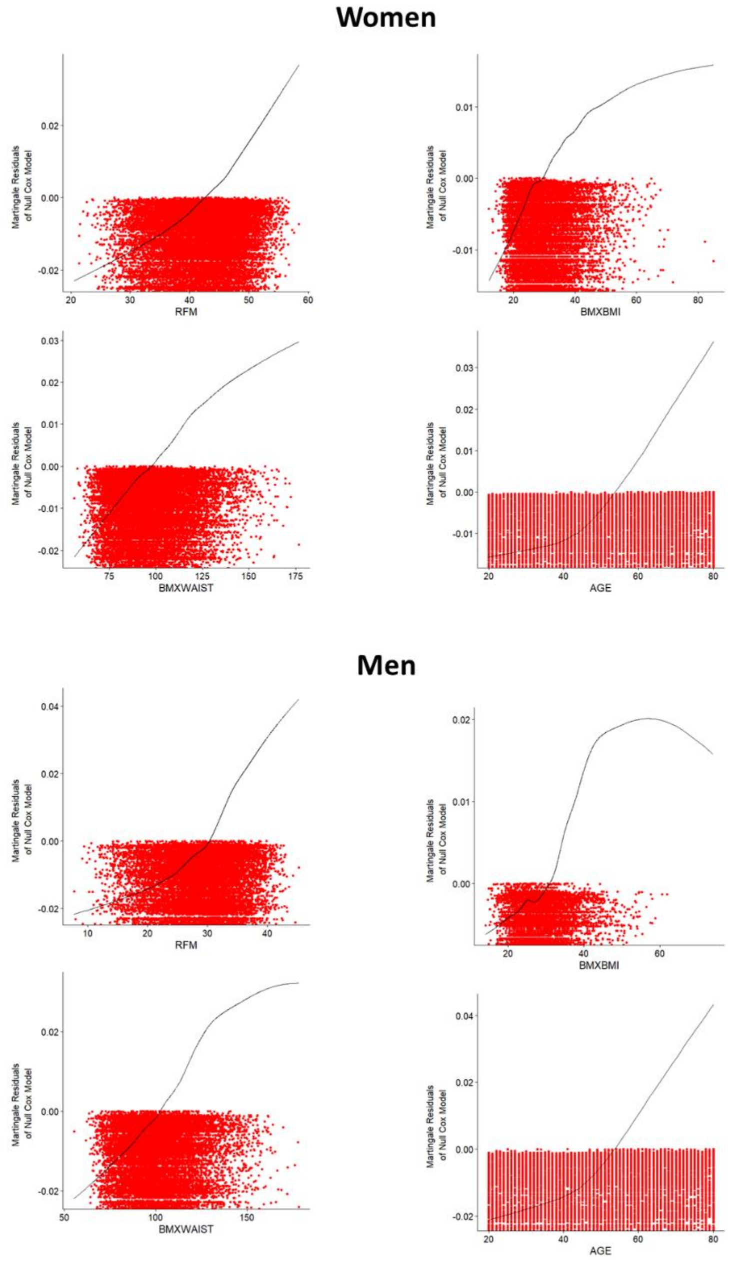

Supplementary Figure S6. Associations of relative fat mass, body mass index, and waist circumference with diabetes-related mortality by quintiles – NHANES 1999-2018. Plots represent the adjusted hazard ratio of diabetes-related mortality using the first weighted quintile of each index as the reference. Models were adjusted for age, ethnicity, education level, and smoking status. Age was modelled as a continuous variable. BMI, body mass index; RFM, relative fat mass; WC, waist circumference.

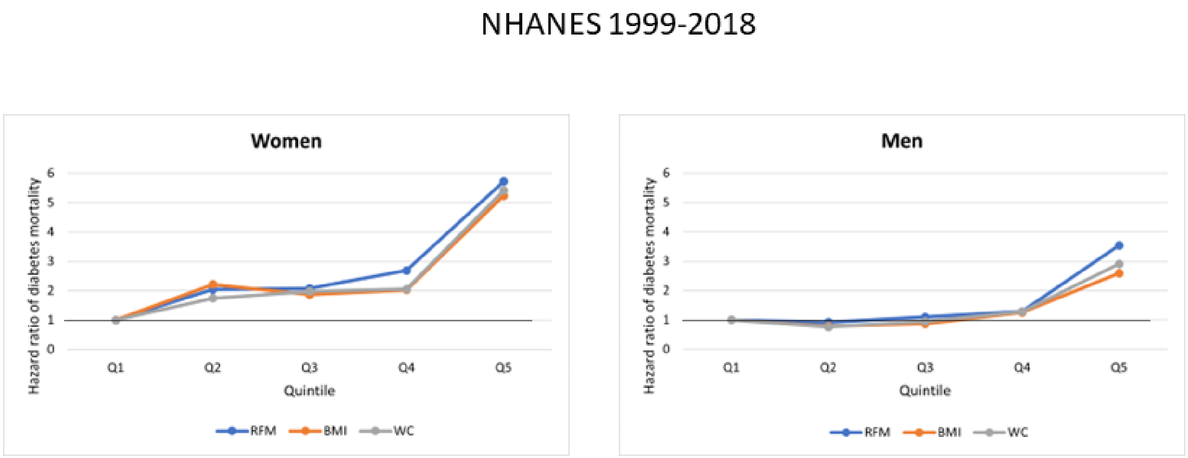

Supplementary Figure S7. Hazard of diabetes-related mortality among participants without chronic disease (heart disease, stroke, cancer, and renal failure) at baseline in NHANES 1999-2018. Plots represent the adjusted hazard, as a measure of risk, of diabetes-related mortality. The curves for women (A) and men (B) represent the adjusted hazard of death times a constant (the inverse of the hazard of diabetes-related mortality at the mean linear predictor). Models were adjusted for age, ethnicity, education level, and smoking status. All anthropometric indexes and age were modelled using restricted cubic splines. Dashed lines represent the 95% confidence intervals. BMI, body mass index; RFM, relative fat mass; WC, waist circumference.

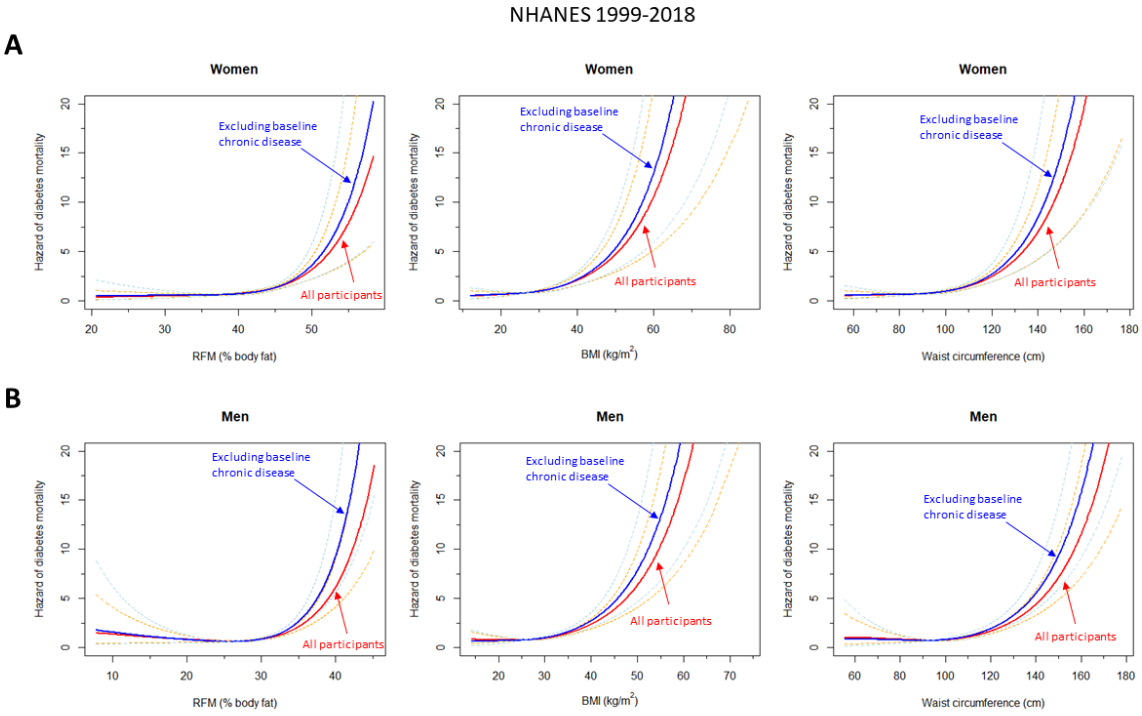

Supplementary Figure S8. Hazard of diabetes-related mortality among participants with a follow-up of at least 3 years in NHANES 1999-2018. Plots represent the adjusted hazard, as a measure of risk, of diabetes-related mortality. The curves for women (A) and men (B) represent the adjusted hazard of death times a constant (the inverse of the hazard of diabetes-related mortality at the mean linear predictor). Models were adjusted for age, ethnicity, education level, and smoking status. All anthropometric indexes and age were modelled using restricted cubic splines. Dashed lines represent the 95% confidence intervals. BMI, body mass index; RFM, relative fat mass; WC, waist circumference.

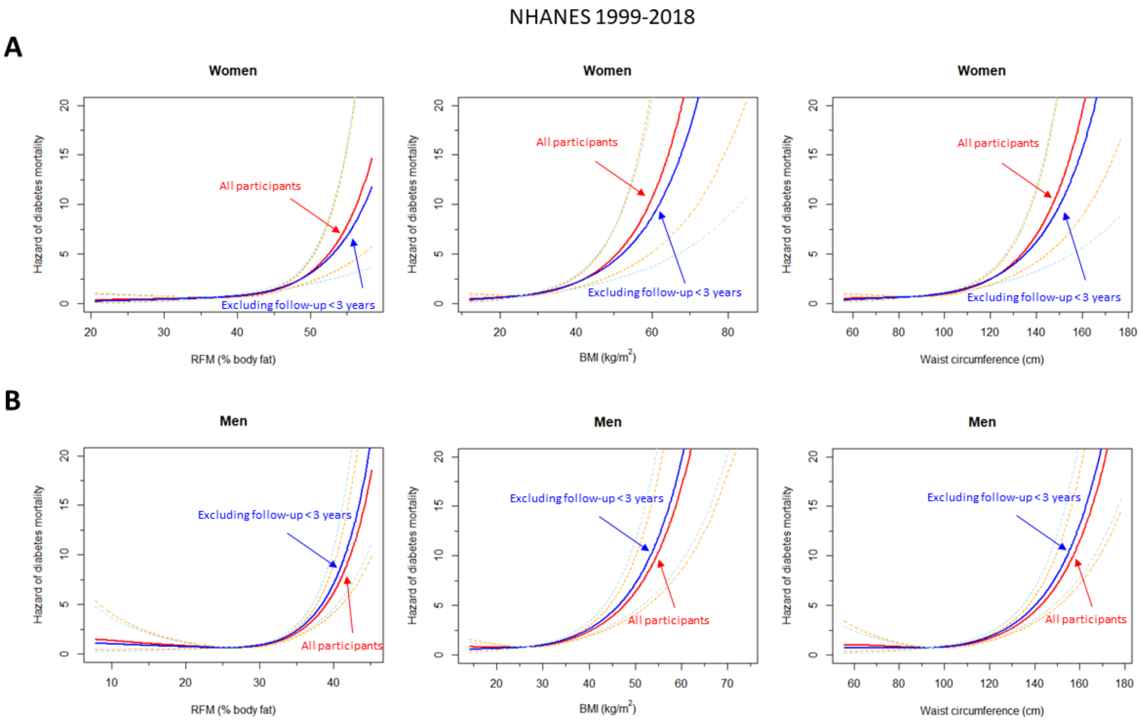

Supplementary Figure S9. Associations of relative fat mass, body mass index, and waist circumference with diabetes-related mortality among never smokers in NHANES 1999-2018. Plots represent the adjusted hazard, as a measure of risk, of diabetes-related mortality for never smokers (if smoked less than 100 cigarettes in life) and for all participants. The curves for women (A) and men (B) represent the adjusted hazard of death times a constant (the inverse of the hazard of diabetes-related mortality at the mean linear predictor). Models were adjusted for age, ethnicity, and education level. All anthropometric indexes and age were modelled using restricted cubic splines. Dashed lines represent the 95% confidence intervals. BMI, body mass index; RFM, relative fat mass; WC, waist circumference.

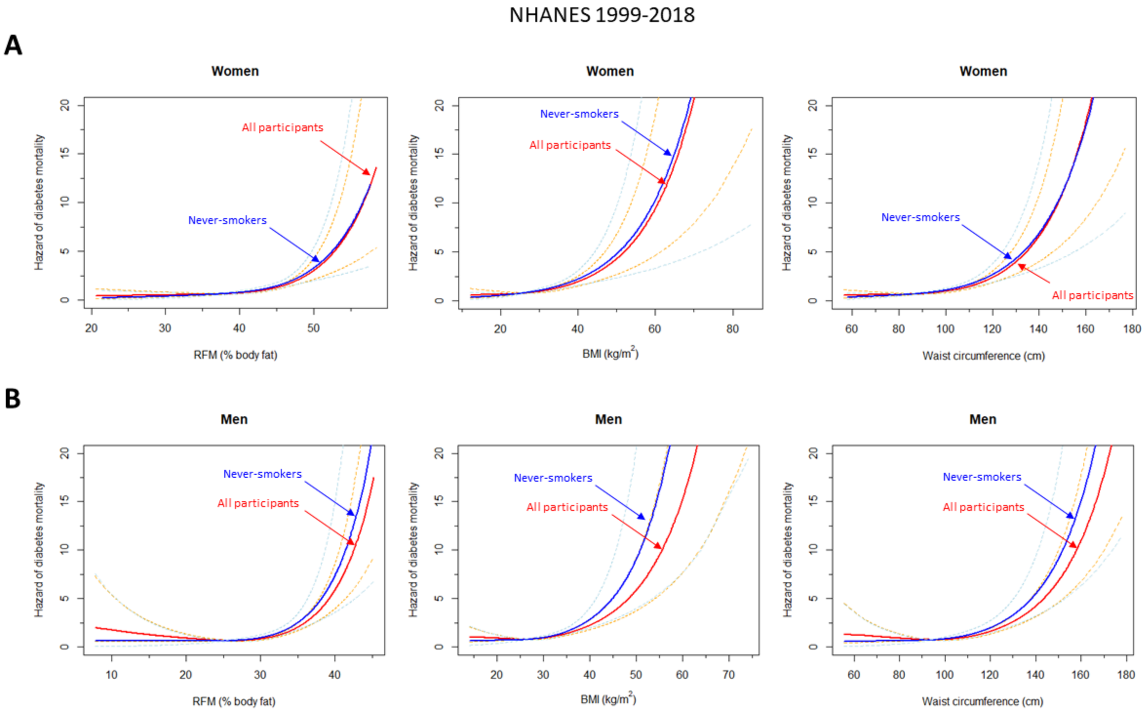

Supplementary Figure S10. Hazard of diabetes-related mortality according to RFM (relative fat mass) by smoking status in men. Plots represent the adjusted hazard, as a measure of risk, of diabetes-related mortality for male current smokers (if smoked at least 100 cigarettes in life and currently smokes) and never/former smokers (those who smoked less than 100 cigarettes in life or were former smokers –if smoked at least 100 cigarettes in life but currently do not smoke). Note that the curves represent the adjusted hazard of death times a constant (the inverse of the hazard of diabetes-related mortality at the mean linear predictor). Models were adjusted for age, ethnicity, and education level. RFM and age were modelled with restricted cubic splines. Among never/former smokers, a hazard ratio can be obtained by dividing the hazard at a given RFM value (e.g., 40%) intersecting the curve for never/former smokers (hazard = ~7) and the hazard at another arbitrary RFM value (e.g., 20%) intersecting the same curve (hazard = ~1). That is, male never/former smokers with an RFM of 40% have a risk seven times greater than the risk of male never/former smokers with an RFM of 20%. Dashed lines represent the 95% confidence interval for current smokers (light blue) and never/former smokers (orange).

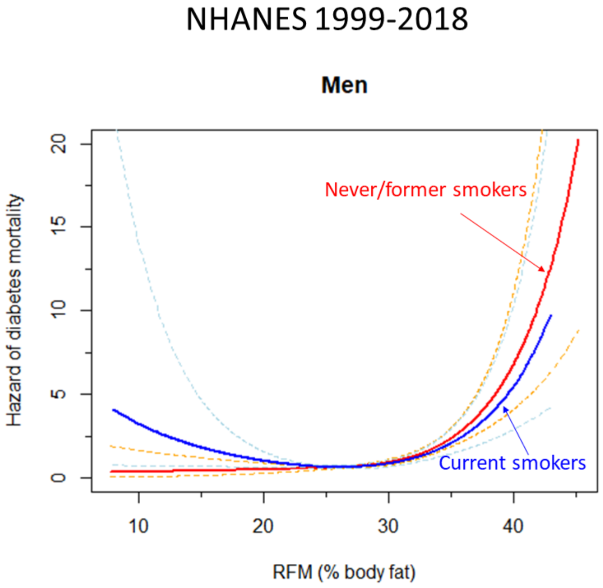

Supplementary Figure S11. Hazard of diabetes-related mortality according to waist circumference values, stratified by age categories in women. Plots represent the adjusted hazard, as a measure of risk, of diabetes-related mortality for women younger than 60 years old and women 60 years of age and older. Note that the curves represent the adjusted hazard of death times a constant (the inverse of the hazard of diabetes-related mortality at the mean linear predictor). Models were adjusted for ethnicity, education level, and smoking status. Waist circumference was modeled using restricted cubic splines. Dashed lines represent the 95% confidence interval for subjects 60 years of age and older (light blue) and younger individuals (orange).

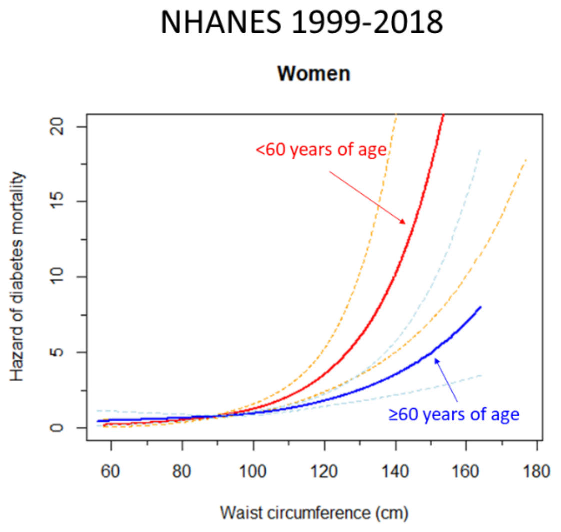

Supplementary Figure S12. Associations of relative fat mass, body mass index, and waist circumference with heart disease mortality in NHANES 1999-2018. Plots represent the weighted adjusted hazard, as a measure of risk, of heart disease mortality for women (A) and men (B). Curves represent the adjusted hazard (not the hazard ratio) of heart disease mortality times a constant (the inverse of the hazard of heart disease mortality at the mean linear predictor). All models were adjusted for age, ethnicity, education level, and smoking status. All anthropometric indexes and age were modelled using restricted cubic splines. Dashed lines represent the 95% confidence intervals. BMI, body mass index; RFM, relative fat mass; WC, waist circumference.

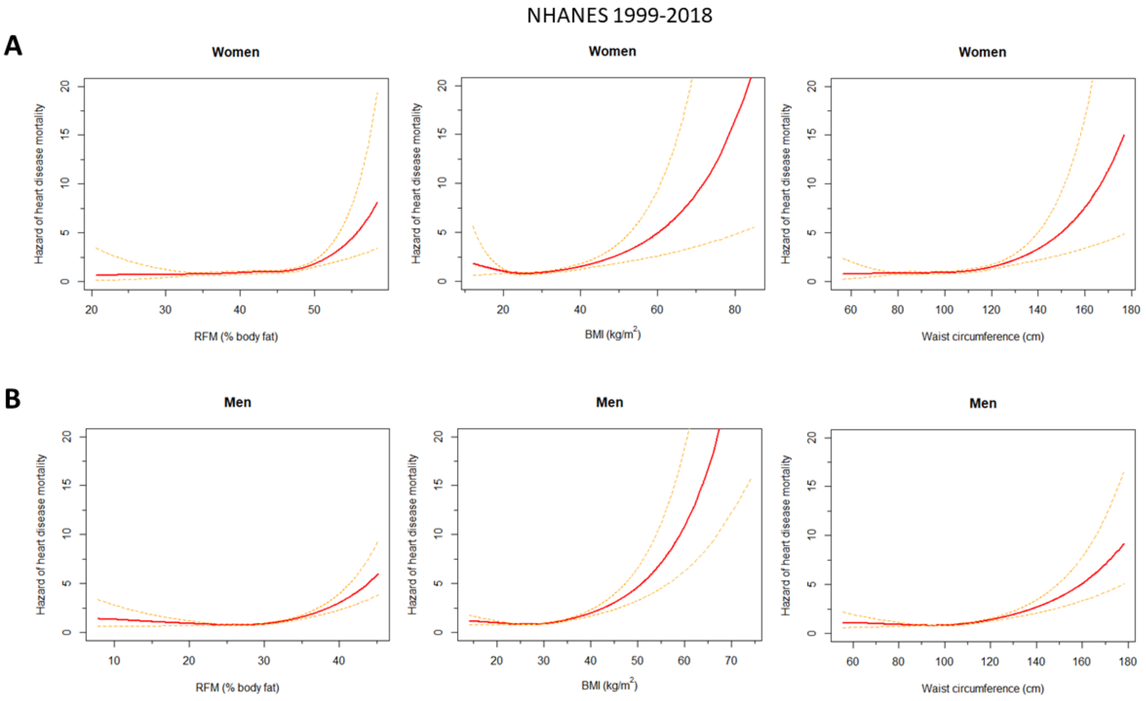

Supplementary Figure S13. Associations of relative fat mass, body mass index, and waist circumference with all-cause mortality in NHANES 1999-2018. Plots represent the weighted adjusted hazard, as a measure of risk, of all-cause mortality for women (A) and men (B). Curves represent the adjusted hazard (not the hazard ratio) of all-cause mortality times a constant (the inverse of the hazard of mortality at the mean linear predictor). All models were adjusted for age, ethnicity, education level, and smoking status. All anthropometric indexes and age were modelled with restricted cubic splines. Dashed lines represent the 95% confidence intervals. BMI, body mass index; RFM, relative fat mass; WC, waist circumference.

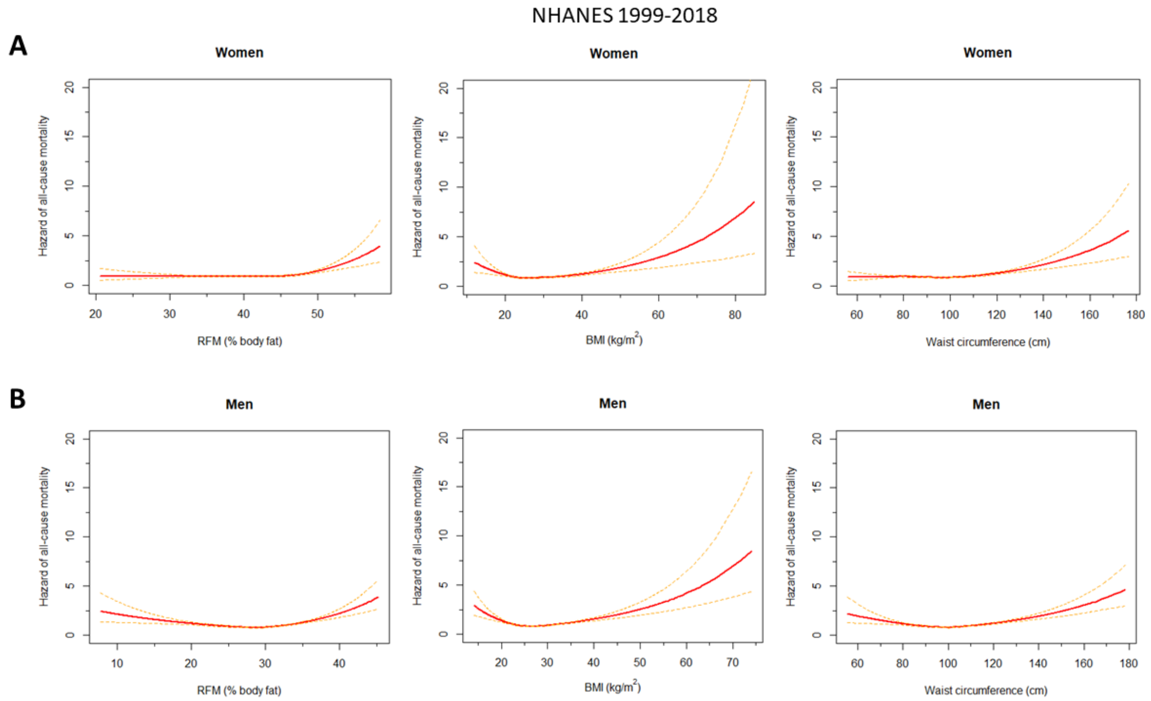

Supplementary Figure S14. Hazard of diabetes-related mortality in NHANES III (1988-1994) according to anthropometric indexes. Plots represent the weighted adjusted hazard of diabetes-related mortality for women (A) and men (B). All models were adjusted for age (as restricted cubic splines), ethnicity, education level, and smoking status. All anthropometric indexes were modelled with restricted cubic splines. Curves represent the adjusted hazard (not the hazard ratio) of diabetes-related mortality relative to a constant. Dashed lines represent the 95% confidence intervals. BMI, body mass index; RFM, relative fat mass; WC, waist circumference.

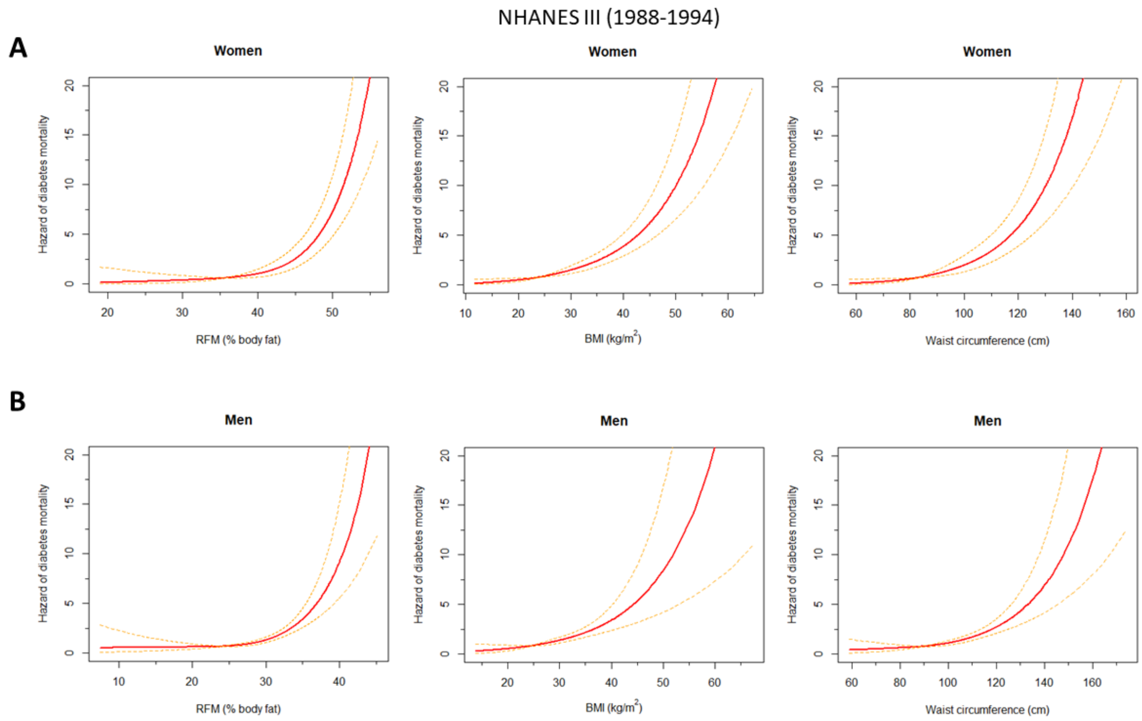

Supplementary Figure S15. Hazard of diabetes-related mortality among participants with a follow-up of at least 3 years in NHANES 1988-1994. Plots represent the adjusted hazard, as a measure of risk, of diabetes-related mortality. The curves for women (A) and men (B) represent the adjusted hazard of death times a constant (the inverse of the hazard of diabetes-related mortality at the mean linear predictor). Models were adjusted for age, ethnicity, education level, and smoking status. All anthropometric indexes and age were modelled using restricted cubic splines. Dashed lines represent the 95% confidence intervals. BMI, body mass index; RFM, relative fat mass; WC, waist circumference.

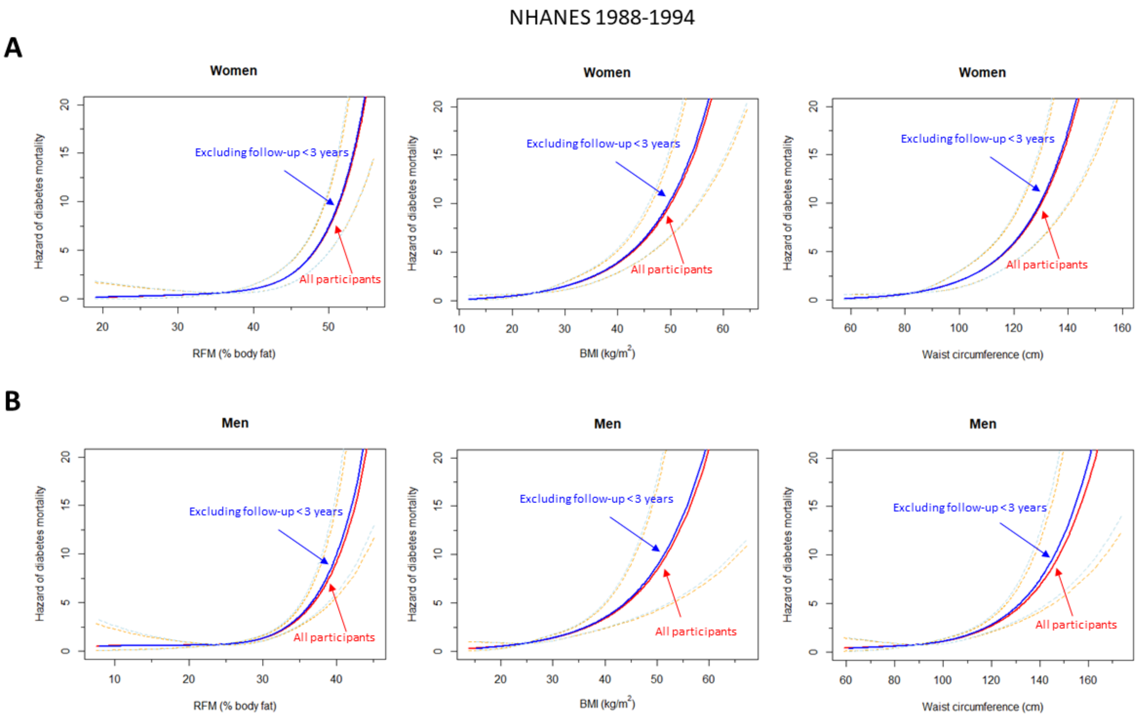

Supplementary Figure S16. Associations of relative fat mass, body mass index, and waist circumference with diabetes-related mortality among participants without heart disease, stroke, cancer, and renal failure at baseline in NHANES III (1988-1994). Plots represent the adjusted hazard, as a measure of risk, of diabetes-related mortality. The curves for women (A) and men (B) represent the adjusted hazard of death times a constant (the inverse of the hazard of diabetes-related mortality at the mean linear predictor). Models were adjusted for age, ethnicity, education level, and smoking status. All anthropometric indexes and age were modelled using restricted cubic splines. Dashed lines represent the 95% confidence intervals. BMI, body mass index; RFM, relative fat mass; WC, waist circumference.

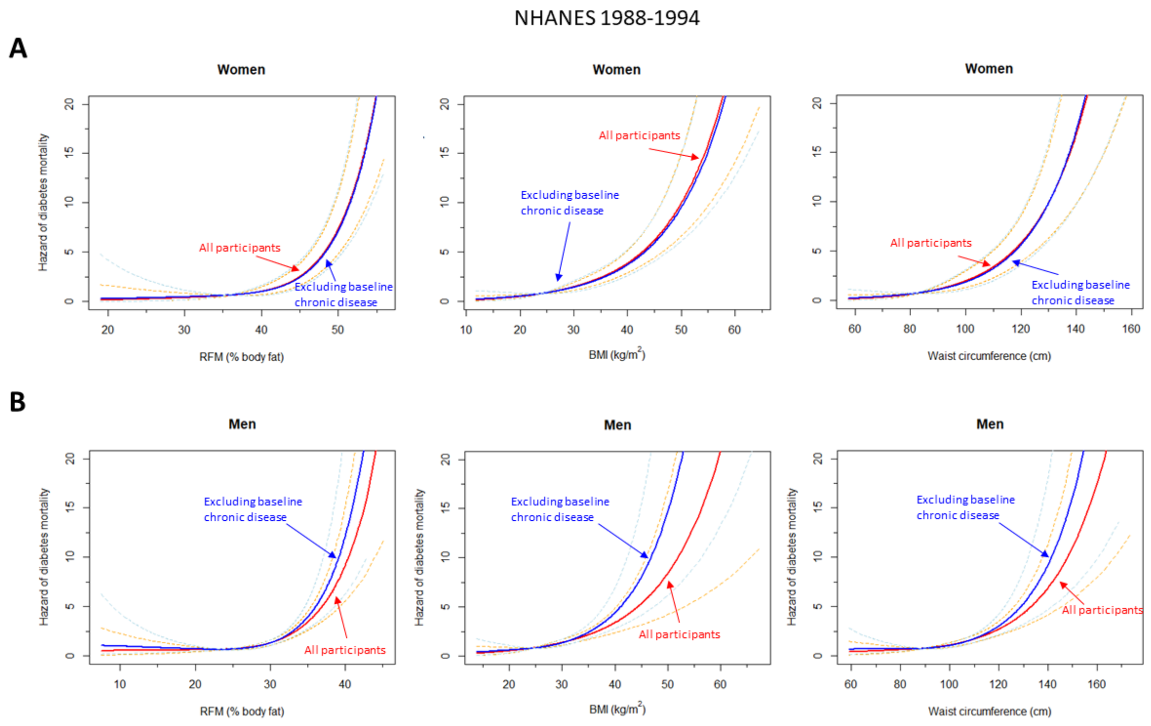

Supplementary Figure S17. Associations of relative fat mass, body mass index, and waist circumference with heart disease mortality in NHANES III (1988-1994). Plots represent the weighted adjusted hazard, as a measure of risk, of heart disease mortality for women (A) and men (B). Curves represent the adjusted hazard (not the hazard ratio) of heart disease mortality times a constant (the inverse of the hazard of heart disease mortality at the mean linear predictor). All models were adjusted for age, ethnicity, education level, and smoking status. All anthropometric indexes and age were modelled with restricted cubic splines. Dashed lines represent the 95% confidence intervals. BMI, body mass index; RFM, relative fat mass; WC, waist circumference.

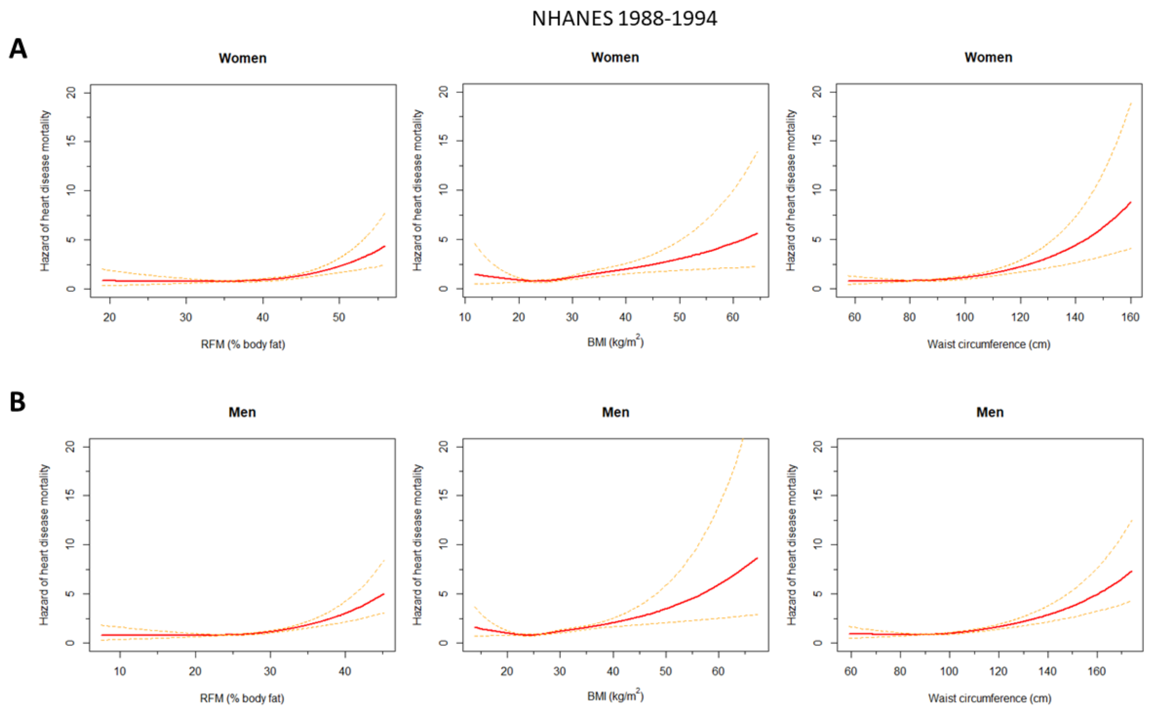

Supplementary Figure S18. Associations of relative fat mass, body mass index, and waist circumference with all-cause mortality in NHANES III (1988-1994). Plots represent the weighted adjusted hazard, as a measure of risk, of all-cause mortality for women (A) and men (B). Curves represent the adjusted hazard (not the hazard ratio) of all-cause mortality times a constant (the inverse of the hazard of all-cause mortality at the mean linear predictor). All models were adjusted for age, ethnicity, education level, and smoking status. All anthropometric indexes and age were modelled with restricted cubic splines. Dashed lines represent the 95% confidence intervals. BMI, body mass index; RFM, relative fat mass; WC, waist circumference.

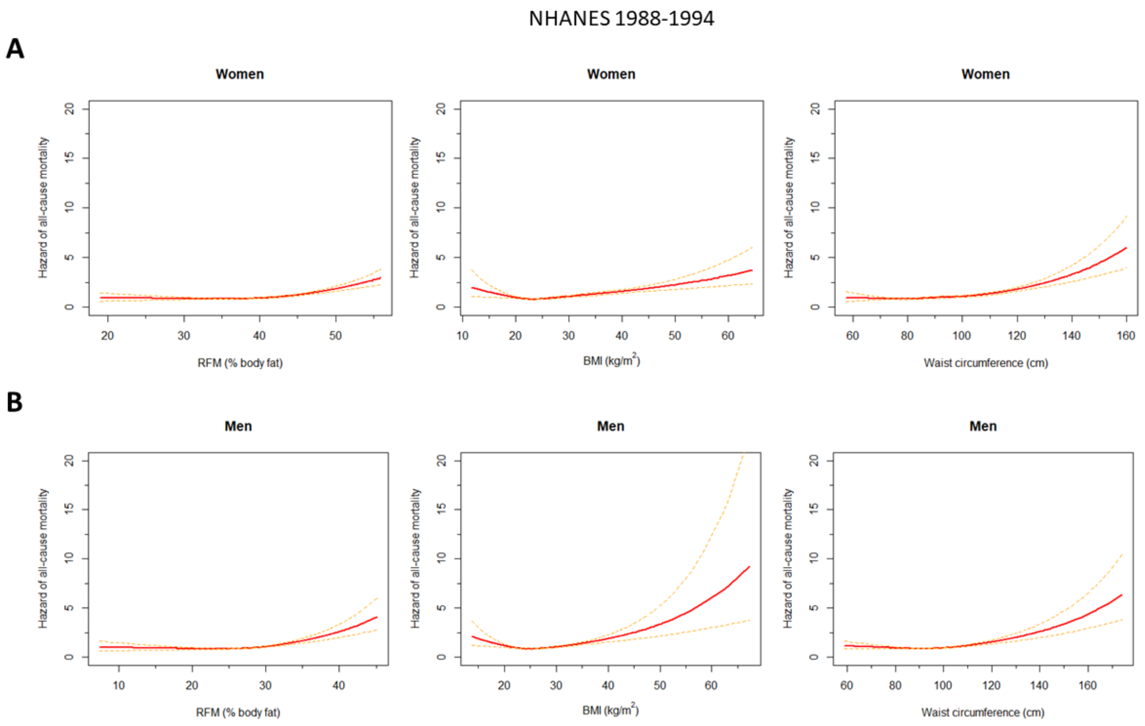

References

1. Curtin LR, Mohadjer LK, Dohrmann SM, et al. The National Health and Nutrition Examination Survey: Sample Design, 1999-2006. *Vital Health Stat 2.* 2012(155):1-39.
2. Johnson CL, Paulose-Ram R, Ogden CL, et al. National health and nutrition examination survey: analytic guidelines, 1999-2010. *Vital Health Stat 2.* 2013(161):1-24.
3. CDC. Cigarette smoking among adults--United States, 1992, and changes in the definition of current cigarette smoking. *MMWR Morb Mortal Wkly Rep.* 1994;43(19):342-346.
4. Levey AS, Coresh J, Greene T, et al. Using standardized serum creatinine values in the modification of diet in renal disease study equation for estimating glomerular filtration rate. *Ann Intern Med.* 2006;145(4):247-254.
5. Woolcott OO, Bergman RN. Defining cutoffs to diagnose obesity using the relative fat mass (RFM): Association with mortality in NHANES 1999-2014. *Int J Obes (Lond).* 2020;44:1301-1310.
6. Schoenfeld D. Partial residuals for the proportional hazards regression model. *Biometrika.* 1982;69(1):239-241.
7. Therneau TM, Grambsch PM, Fleming TR. Martingale-based residuals for survival models. *Biometrika.* 1990;77(1):147-160.
8. Akaike H. A new look at the statistical model identification. *IEEE Transactions on Automatic Control.* 1974;19(6):716-723.
9. Bozdogan H. Model selection and Akaike's Information Criterion (AIC): The general theory and its analytical extensions. *Psychometrika.* 1987;52(3):345-370.
10. Harrell FE. *Regression modeling strategies: With applications to linear models, logistic regression, and survival analysis.* New York: Springer; 2001.
11. R Core Team. R: A language and environment for statistical computing. 2023.
12. Durrleman S, Simon R. Flexible regression models with cubic splines. *Stat Med.* 1989;8(5):551-561.
